# Spatially Resolved Transcriptomes of Mammalian Kidneys Illustrate the Molecular Complexity and Interactions of Functional Nephron Segments

**DOI:** 10.3389/fmed.2022.873923

**Published:** 2022-07-07

**Authors:** Arti M. Raghubar, Duy T. Pham, Xiao Tan, Laura F. Grice, Joanna Crawford, Pui Yeng Lam, Stacey B. Andersen, Sohye Yoon, Siok Min Teoh, Nicholas A. Matigian, Anne Stewart, Leo Francis, Monica S. Y. Ng, Helen G. Healy, Alexander N. Combes, Andrew J. Kassianos, Quan Nguyen, Andrew J. Mallett

**Affiliations:** ^1^Kidney Health Service, Royal Brisbane and Women's Hospital, Herston, QLD, Australia; ^2^Conjoint Internal Medicine Laboratory, Chemical Pathology, Pathology Queensland, Health Support Queensland, Herston, QLD, Australia; ^3^Faculty of Medicine, University of Queensland, Brisbane, QLD, Australia; ^4^Anatomical Pathology, Pathology Queensland, Health Support Queensland, Herston, QLD, Australia; ^5^Institute for Molecular Bioscience, University of Queensland, Brisbane, QLD, Australia; ^6^School of Biomedical Sciences, The University of Queensland, Brisbane, QLD, Australia; ^7^Genome Innovation Hub, University of Queensland, Brisbane, QLD, Australia; ^8^UQ Sequencing Facility, Institute for Molecular Bioscience, University of Queensland, Brisbane, QLD, Australia; ^9^UQ Diamantina Institute, Faculty of Medicine, The University of Queensland, Woolloongabba, QLD, Australia; ^10^QCIF Facility for Advanced Bioinformatics, Institute for Molecular Bioscience, The University of Queensland, Brisbane, QLD, Australia; ^11^Nephrology Department, Princess Alexandra Hospital, Woolloongabba, QLD, Australia; ^12^Department of Anatomy and Developmental Biology, Stem Cells and Development Program, Monash Biomedicine Discovery Institute, Monash University, Melbourne, VIC, Australia; ^13^College of Medicine & Dentistry, James Cook University, Townsville, Queensland, QLD, Australia; ^14^Department of Renal Medicine, Townsville University Hospital, Townsville, Queensland, QLD, Australia

**Keywords:** spatial transcriptomics, kidney, human, mouse, cell-cell interactions

## Abstract

Available transcriptomes of the mammalian kidney provide limited information on the spatial interplay between different functional nephron structures due to the required dissociation of tissue with traditional transcriptome-based methodologies. A deeper understanding of the complexity of functional nephron structures requires a non-dissociative transcriptomics approach, such as spatial transcriptomics sequencing (ST-seq). We hypothesize that the application of ST-seq in normal mammalian kidneys will give transcriptomic insights within and across species of physiology at the functional structure level and cellular communication at the cell level. Here, we applied ST-seq in six mice and four human kidneys that were histologically absent of any overt pathology. We defined the location of specific nephron structures in the captured ST-seq datasets using three lines of evidence: pathologist's annotation, marker gene expression, and integration with public single-cell and/or single-nucleus RNA-sequencing datasets. We compared the mouse and human cortical kidney regions. In the human ST-seq datasets, we further investigated the cellular communication within glomeruli and regions of proximal tubules–peritubular capillaries by screening for co-expression of ligand–receptor gene pairs. Gene expression signatures of distinct nephron structures and microvascular regions were spatially resolved within the mouse and human ST-seq datasets. We identified 7,370 differentially expressed genes (*p*_adj_ < 0.05) distinguishing species, suggesting changes in energy production and metabolism in mouse cortical regions relative to human kidneys. Hundreds of potential ligand–receptor interactions were identified within glomeruli and regions of proximal tubules–peritubular capillaries, including known and novel interactions relevant to kidney physiology. Our application of ST-seq to normal human and murine kidneys confirms current knowledge and localization of transcripts within the kidney. Furthermore, the generated ST-seq datasets provide a valuable resource for the kidney community that can be used to inform future research into this complex organ.

## Introduction

The mammalian kidney contains millions of nephrons, each composed of functional structures including the distal tubule, the loop of Henle, the proximal tubule, and the glomerulus. Nephrons are connected to a collecting duct network and surrounded by stroma and microvasculature (1, 2). The nephrons maintain homeostasis of body fluids, electrolyte and acid–base balance, and the excretion of metabolic waste products (3–5). The spatial organization of nephrons facilitates the homeostatic function of the mammalian kidney. However, to date, transcriptome studies of normal human and murine nephrons have utilized bulk RNA-sequencing, single-cell and/or single-nucleus RNA-sequencing (scRNA-seq/snRNA-seq), which require manipulation of tissue, including tissue homogenization or cell dissociation and resulting in the loss of crucial spatial information (6–13).

Unlike bulk RNA-seq, scRNA-seq, and snRNA-seq, ST-seq provides crucial spatial information with transcriptome profiling by integrating histology with RNA-seq within intact tissue (14–32). Both histological assessment and RNA-seq are completed sequentially on the same tissue section placed on a glass slide with printed oligo-dT spots, termed ST-spots (14, 17, 33, 34). Transcriptomes within the tissue section are captured by the underlying ST-spots and receive a spatial barcode in the process. The sequenced ST-spot transcriptomes are subsequently aligned with the Hematoxylin and Eosin (H&E) image to visualize gene expression within the intact tissue. Current applications of ST-seq in mammalian kidneys have been limited to inflammatory or developmental murine models, with no to minimal studies in normal/control mouse and human kidneys (6–9).

In this study, we used a commercially available 10x Genomics ST platform to investigate spatially resolved gene expression in normal mouse and human kidney tissues. We generated transcriptional profiles of the mammalian kidney to identify functional nephron structures and major cell types. Next, we used the generated ST-seq data to investigate differences in gene expression and biological processes between cortical regions of mouse and human kidneys. Last, we predicted cell-cell interactions within glomeruli and regions of proximal tubules–peritubular capillaries (PT–PC). We found that the generated spatial transcriptomic data from normal human and murine kidneys matched current knowledge and localization of transcripts. The generated ST-seq datasets are a valuable data resource for the kidney community to inform future research into this complex organ.

## Materials and Methods

### Kidney Tissue Samples

**Figure 1:**
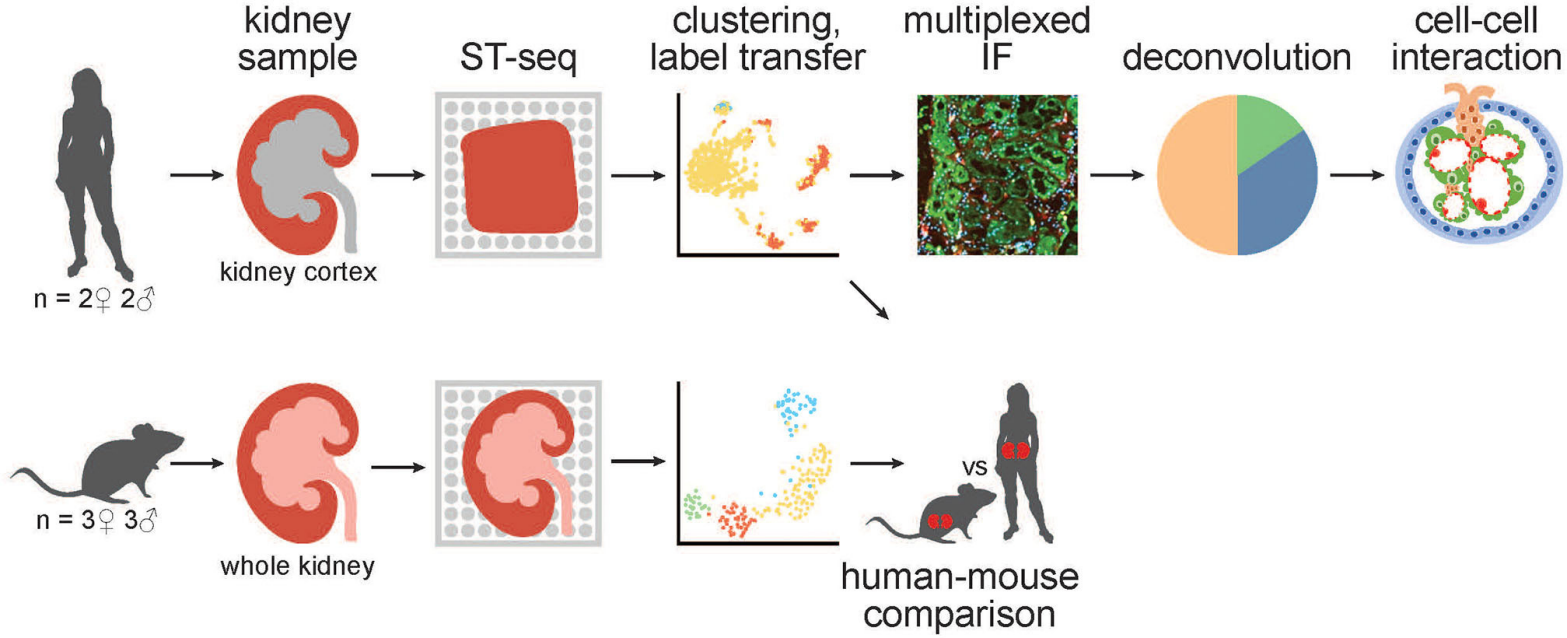
A schematic of the workflow for generation and analysis of mammalian kidney ST-seq datasets. (From left to right) We performed ST-seq on four human kidney cortical tissues (2 women, 51–53 years old and 2 men, 54 and 56 years old) and six mice whole kidneys (3 males, 8 weeks old and 3 females, 6 weeks old). With the generated ST-seq datasets, we performed clustering and label transfer to define the location of specific nephron segments or regions in human and mouse kidneys, respectively. We selected the cortical kidney regions in the mouse ST-seq datasets and performed functional analysis on genes that were differentially expressed between species in the cortex. In the human cortical kidney tissues, we performed multiplexed IF on consecutive deeper sections to correlate the label transfer annotation of the functional nephron segments with histomorphology. Last, we investigated the CCI by screening for L–R gene pair co-expression in glomerular and PT–PC ST-spots identified by deconvolution.

**Figure 2:**
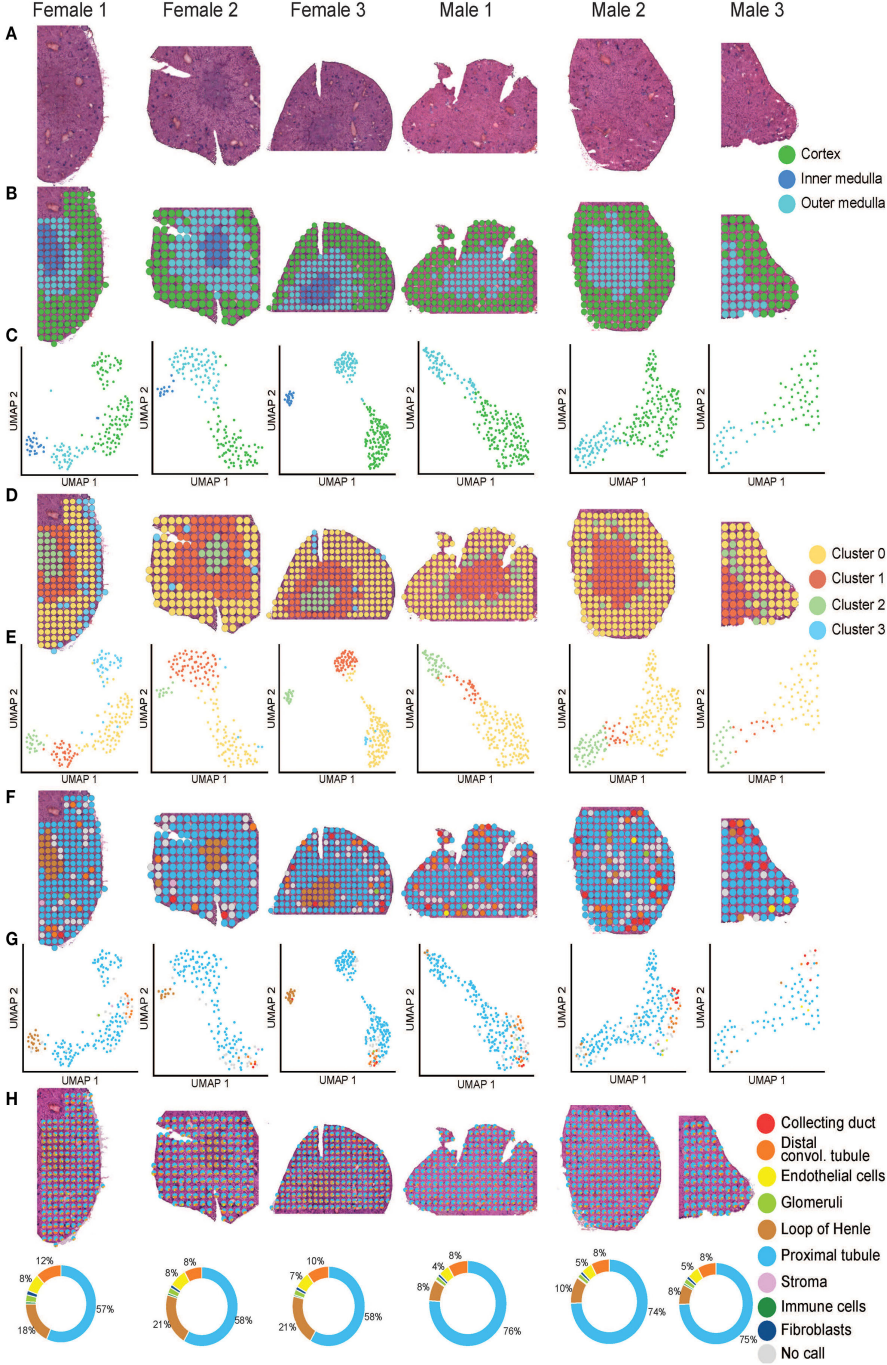
Mouse ST-seq consensus labels. **(A)** H&E images of the mouse kidney tissues from three females and three males. **(B, C)** The functional cortical and medullary regions, which were annotated within the mouse ST-seq datasets by a Consultant Pathologist were mapped to the H&E tissue sections and presented in the UMAP. **(D, E)** The spatial organization of the KNN clusters was mapped to the H&E tissue images and presented in a UMAP. **(F,G)** The spatial organization of the consensus-based label transfer results was mapped to the H&E tissue images and presented in a UMAP, respectively. **(H)** The spatial organization of the deconvoluted functional structures was mapped to the H&E tissue images and presented as simple pie charts to demonstrate the proportions.

Whole mouse kidneys utilized in this ST study were from three male (8 weeks old) and three female (6 weeks old) C57BL/6J wild-type mice (Animal Ethics Committee approval UQDI/452/16 and IMB123/18). The mouse kidneys were collected during tissue harvesting and snap frozen in standard biopsy cryomolds (Tissue-Tek, Sakura Finetek, United States) with optimum cutting temperature (OCT) compound (Tissue-Tek). These freshly frozen adult mouse kidneys were then stored at −80°C on site. Cryosections of 10 μm were cut from the mouse samples, stained with H&E, and confirmed as normal by a Consultant Pathologist. These samples were subsequently used for ST-seq with the ST platform (100 μm ST-spots; Figures 1, 2A).

**Figure 3:**
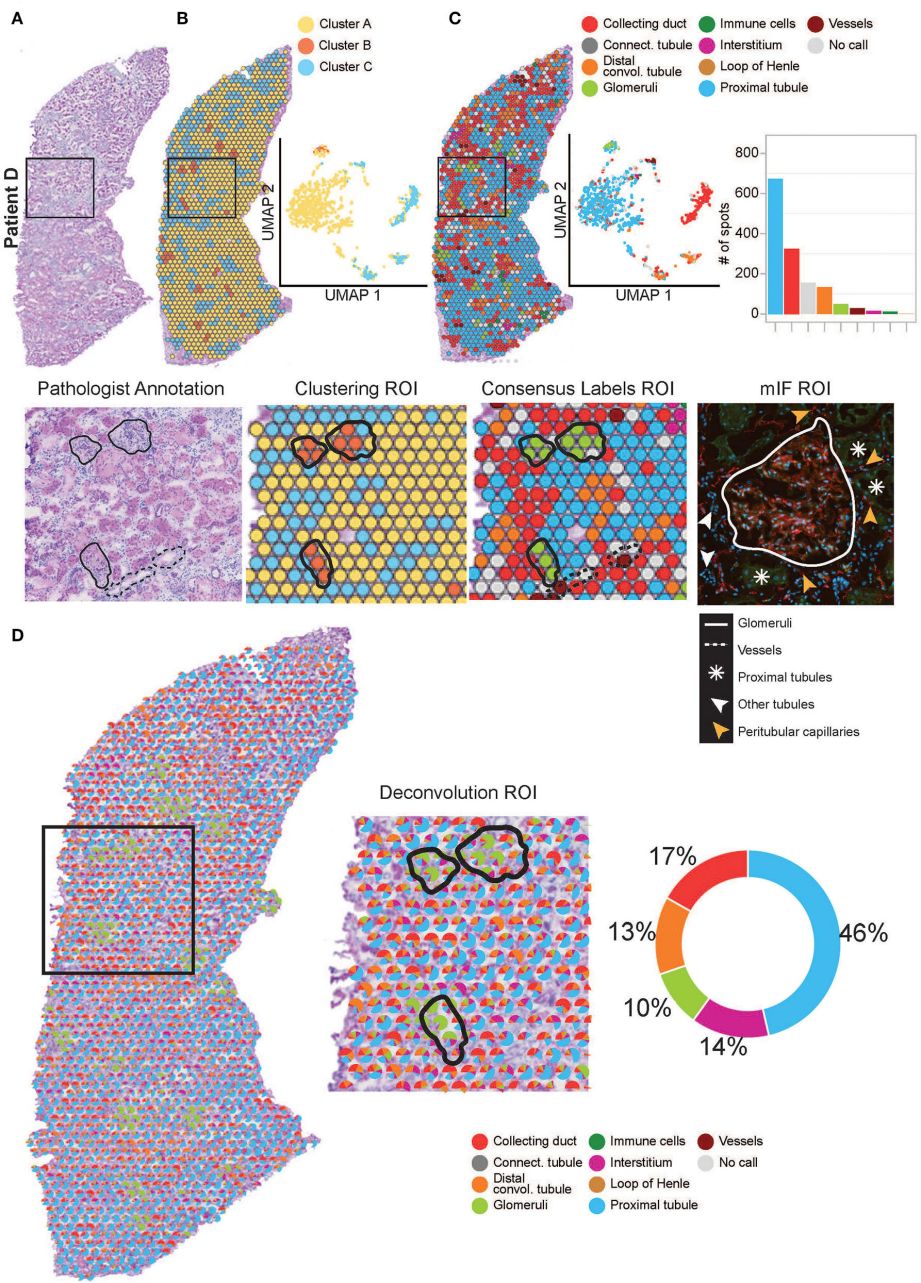
Annotation of functional structures within patient D. **(A)** H&E image and a zoomed-in region of interest (ROI) of the pathologist's annotation of glomeruli and large vasculature. **(B)** The spatial organization of the KNN clusters was mapped to the H&E tissue images, presented in a UMAP and a zoomed-in ROI of clustering. **(C)** The spatial organization of the consensus-based label transfer results was mapped to the H&E tissue images, in a UMAP, a simple bar chart, and a zoomed-in ROI of consensus-based label transfer. A zoomed-in ROI stained with mIF (red = anti-CD31 for endothelial cells, green = anti-AQP1 for proximal tubule cells, and blue = DAPI for nuclei) demonstrates the abutting nature of functional structures within the cortical kidney tissue. **(D)** Further deconvolution demonstrates the distribution and proportions of functional structures within the cortical kidney tissue which are mapped to the H&E image, presented as a simple pie chart and a zoomed-in ROI. For the annotation of functional structures within patients A, B, and C cortical kidney tissue, ***see*** Supplementary Figure 1.

We utilized human cortical kidney tissues taken a minimum of 3 cm away from the tumor margins of four patients that were matched for comorbidities (2 women, 51–53 years old and 2 men, 54 and 56 years old; Table 1). The use of human kidney tissues was approved by the Royal Brisbane and Women's Hospital Human Research Ethics Committee (2002/011). Human kidney tissue was snap frozen in standard biopsy cryomolds (Tissue-Tek) with OCT compound (Tissue-Tek). Cryosections of 10 μm were cut from the human kidney samples, stained with H&E, and confirmed as normal by a Consultant Pathologist. These samples were subsequently used for ST-seq with the Visium ST platform (55 μm ST-spots; Figures 1, 3A and Supplementary Figure 1A).

**Table 1:** Patient cohort characteristics.

**Patient ID**	**A**	**B**	**C**	**D**
**Age (years)/ gender (M/F)**	51/F	54/M	53/F	56/M
**eGFR (mL/min/1.73m** ^**2**^ **)**	>90	88	89	86
**Serum creatinine (mmol/L)**	50	86	68	86
**Pathology**	ccRCC	ccRCC	ccRCC	ccRCC
**Metastasis**	neg	neg	neg	neg
**Co-morbidities**				
**Hypertension**	neg	neg	neg	neg
**Smoker**	neg	neg	neg	yes
**Coronary artery disease**	neg	neg	neg	neg
**Peripheral vascular disease**	neg	neg	neg	neg
**Diabetes mellitus**	neg	neg	neg	neg
**Hepatitis B and C**	neg	neg	neg	neg

*Key, ccRCC is clear cell renal cell carcinoma*.

### RNA Quality

Two 10 μm scrolls of tissue were collected in pre-chilled 1.5 ml Eppendorf tubes from each frozen OCT block of mouse whole kidneys (*n* = 6) and human cortical kidneys (*n* = 4). RNA from each sample was extracted from the cryosectioned scrolls according to the QIAGEN RNeasy micro kit (Hilden, Germany). RNA content was quantified according to the Qubit RNA HS assay kit (Invitrogen, Thermo Fisher Scientific, Singapore) and the RNA integrity number (RIN) was assessed according to the Agilent 2100 Bioanalyzer RNA 6000 Pico assay (Agilent Technologies, Inc., United States). The measured RINs for all kidney tissues were >7.

### Tissue Optimization

Tissue optimization was performed according to the 10x Genomics ST Tissue Optimization Manual (version 190219, 10x Genomics, United States) to determine the ideal permeabilization time. Frozen 10 μm cryosectioned tissue from mouse and human kidney tissues were utilized for this optimization. The kidney tissue sections were dried at 37°C for 1 min, fixed in pre-chilled 100% methanol at −20°C for 30 min, and stained in Mayer's Hematoxylin (Dako, Agilent Technologies, Inc., United States) for 5 min and Eosin (Sigma–Aldrich Pty. Ltd., Australia) for 2 min. Imaging was performed on an Aperio XT brightfield slide scanner (Leica).

After H&E imaging, the kidney tissue sections were placed in a permeabilization mix over a range of time points to allow the mRNA to drop down from the tissue sections and bind to the oligo-dTs printed on the slide. The captured mRNAs on the slide surface were then reverse transcribed to fluorescently labeled cDNA. This fluorescent cDNA signal was imaged on a Leica confocal microscope (SP8 STED 3X). The ideal permeabilization time of 12 min was determined by comparing both the H&E and fluorescent images from the tissue optimization slide. This optimized permeabilization time was utilized for generating ST libraries for sequencing from mouse and human kidney tissue sections.

### Library Preparation

ST library preparation of the mouse kidney tissues (*n* = 6) was performed according to the ST Library Preparation Manual (version 190219, 10x Genomics, United States). ST library preparation of the human cortical kidney tissues (*n* = 4) was performed according to the Visium Spatial Gene Expression Reagent Kits User Guide (CG000239 Rev C, 10x Genomics, United States). In brief, 10 μm cryosectioned mouse and human kidney tissues were placed onto pre-chilled library preparation slides. The mouse kidneys were multiplexed into two arrays based on gender (three mouse kidneys per array). Sections of the human kidney were placed in four separate arrays such that each patient received an individual array. We placed two consecutive sections in arrays A and D. Tissue sections were dried on the slides at 37°C for 1 min, then fixed in pre-chilled 100% methanol at −20°C for 30 min, and stained in Mayer's Hematoxylin for 5 min and Eosin for 2 min. Brightfield imaging was performed on an Axio Z1 slide scanner (Zeiss). Based on the shorter (539–683 bp) cDNA libraries generated from the human cortical kidney tissue sections, we reduced the fragmentation reaction and the SPRI bead ratio from the manufacturer's recommendation. To further remove smaller library insert sizes, we gel extracted the library preparations for patients A, B, and C, followed by DNA clean-up according to the Monarch PCR and DNA clean-up kit (New England BioLabs). All libraries were loaded at 1.8 pM. Libraries from patients A, B, and C, and mice kidneys were sequenced using a High output reagent kit (Illumina). Library from patient D was sequenced using a Mid output reagent kit (Illumina) on a NextSeq500 (Illumina) instrument. Sequencing was performed using the following protocol: Read1–28bp, Index1–10bp, Index2–10bp, Read2–120bp.

### ST-Seq Data Processing and Mapping

Illumina generated ST-seq libraries were first converted from raw base call (BCL) files to FASTQ files using bcl2fastq/2.17. Complex ST-seq libraries were retained and the FASTQ reads were trimmed of poly-A sequences on the 3' end and TSO sequences on the 5' end using cutadapt/1.8.3 (35). The cleaned FASTQ files were then mapped by Space Ranger V1.0 (10x Genomics) to the mouse reference genome and gene annotations (GRCm38–mm10) or human reference genome and gene annotations (GRCh38–3.0.0). The captured genes were mapped to the spatial coordinates across the H&E image obtained during the library preparation based on the detection of the tissue area and the alignment to fiducial markings. The multiplexed mouse ST-seq datasets were extracted to individual tissue sections using Loupe Browser (v4.0, 10x Genomics, United States).

We collectively detected more than 22,000 genes (GRCm38 –mm 10) across 1,160 ST-spots within the mouse ST-seq datasets. The median number of genes per spot ranged from 3,310 to 5,994 while median UMIs captured per spot spanned 10,491–31,145 (Supplementary Figure 2A). Within the human ST-seq datasets, we collectively detected over 23,000 genes (GRCh38-3.0.0) across 4,918 ST-spots. The median number of genes per spot ranged from 674 to 1,519, while the median unique molecular identifiers (UMIs) captured per spot spanned from 1,139 to 3,037 (Supplementary Figure 3A).

### Spatial Analysis Using a Seurat Analytical Pipeline

Both mouse and human ST-seq datasets were analyzed using Seurat v4 (36–39). Preliminary quality control steps involved the filtration of ST-spots containing more than 50% mitochondrial genes (mtRNA) or 50% ribosomal genes (rbRNA). No ST-spots reached this rbRNA threshold. In the mouse ST-seq datasets, the level of mtRNA expression was consistently below 20% (Supplementary Figure 2B). However, high levels (median ~ 12–28% total reads) of mtRNA expression were observed in the human ST-seq datasets (Supplementary Figure 3B). Thus, we used a threshold to filter only those ST-spots where mtRNA represented less than 50% of total reads for the human ST-seq datasets. Visual inspection of the mtRNA distribution in human kidney tissue sections with filtering (Supplementary Figures 3C,D) and the mouse kidney tissue sections with no filtering (Supplementary Figure 2C) showed a similar mtRNA expression pattern.

The top 2,000 most variable genes across ST-spots were detected by Seurat and were normalized using Scran before running principal component analysis (40, 41). Uniform manifold approximation and projection (UMAP) dimensionality reduction and clustering were performed using the top 50 principal components (42). Clustering was tested using a range of resolution values from 0.1 to 1.6, and the highest average stable resolution value was selected for each sample using the SC3 stability measure from Clustree (43). The generated clustering results were visualized in both two-dimensional UMAP space and spatial context mapped over the H&E images.

We performed label transfer in two sequential steps using a collection of publicly available snRNA-seq and/or scRNA-seq kidney datasets to predict cell types (Supplementary Tables 1, 2). This label transfer method projects existing reference datasets and new datasets with unknown cell types (query) into a shared low-dimensional space. The equivalent cell types (or anchor cell types) are arranged in the same neighborhood thus, allowing for inference of cell types in the new query datasets from the reference datasets. For each query cell type, a confidence score (scaled 0 to 1) was calculated based on the shared neighbor information with the reference cell type. First, label transfer annotation from mouse scRNA-seq and human snRNA-seq reference datasets was used to determine high-confidence ST-spot annotations. In the second round, mouse and human scRNA-seq reference datasets were used to label the remaining unlabeled ST-spots (Supplementary Figures 4, 5). In both rounds, the transfer of cell-type annotations from the reference to a query ST-spot was made if the confidence score for the top match was >0.6.

### Differential Gene Expression Analysis Within the Cortical Regions Between Species

We focused the differential gene expression analysis on the 708 cortical kidney ST-spots in the mouse ST-seq datasets. Raw gene expression counts were first aggregated by tissue samples to remove potential technical variation between intra-sample ST-spots and to account for species as two conditions and samples as biological replicates (44). The aggregation was performed using aggregateAcrossCells() function in Scater package and then normalized by library size, using sample-specific normalization factors calculated by the function calcNormFactors() in edgeR package (45, 46). Each tissue sample was treated as pseudo-bulk data to fit in a gene-wise linear model glmQLFit(), which estimates quasi-likelihood dispersions across species (conditions) and samples (biological replicates). We then implemented empirical Bayes quasi-likelihood F-tests in the glmQLFTest() function to identify differentially expressed genes (all genes with an FDR <0.05 and no log-fold change cut-off).

### Deconvolution at the Functional Structure and Cell-Type Level

Deconvolution compares the expression profile from thousands of genes detected in each ST-spot to the expression patterns of cell type–specific marker genes within the reference datasets, to predict the proportion of different functional structures present in each ST-spot. We identified the proportion of specific cell types within each ST-spot using robust cell-type decomposition (RCTD)—a method that accounts for technical variation between different technologies, (47). In both mouse and human ST-seq datasets, we completed deconvolution to the functional structure level. In the human ST-seq datasets, we selected the ST-spots that were deconvoluted at the functional level as glomerular, proximal tubular, and peritubular capillaries for further deconvolution to cell-type level to perform cell-cell interaction (CCI) analysis.

### StLearn Cell-Cell Interaction Analysis Within the Human ST-Seq Datasets

Cell-cell interaction analysis was performed using stLea “rn to predict interactions between spots or within each spot (48). “Between-spot” mode tests for significantly enriched CCI scores between any given ST-spot and its adjacent neighbors within the tissue, while “within-spot” mode tests for significantly enriched CCI scores within each ST-spot itself as multiple cells could be present within the each ST-spot. Briefly, there are four main steps in the CCI analysis. Step 1: CCI identifies cell-type diversity across the tissue. Step 2: CCI identifies L–R co-expression (CCI–LR) between or within spots for every ST-spot underlying the tissue. We used connectomeDB for the human ST-seq datasets (49). Step 3: The cell-type diversity score CCI–HET spot and CCI–LR spot score are standardized to unit variance and multiplied to form composite CCI scores that account for both cell-type diversity and the level of local co-expression values for each L–R pair. A high CCI score for an L–R pair indicates tissue areas that are most likely to harbor active CCI of the pair. Step 4: A negative binomial model is fitted to a null distribution of CCI scores calculated for thousands of random pairings of non-interacting protein–protein pairs. The best fit model is then used to statistically test for significance of discovering highly interacting spots, by calculating the probability of observing a CCI score for a given L–R pair given the null distribution.

### Multiplex Immunofluorescence Staining

Consecutive deeper 10 μm cryosections from the human cortical kidney tissues (*n* = 4) used for ST-seq were placed onto room temperature SuperFrost Ultra Plus slides (Thermo Scientific, United States). The tissue sections were then adhered to the slides by drying for 1 min at 37°C and fixed with pre-chilled 100% methanol at−20°C for 30 min. Non-Specific binding was blocked with 10% donkey serum (Merck–Millipore, Burlington, MA, United States) for 15 min. Sections were incubated in a primary antibody mix comprising anti-endothelial cells (monoclonal mouse anti-human CD31; Clone JC70A; Dako Omnis) and anti-Aquaporin-1 (polyclonal rabbit anti-human AQP1 (H-55); SC-20810; Santa Cruz Biotechnology) for 20 min. Fluorescent labeling was obtained with AlexaFluor-conjugated secondary antibodies [donkey anti-mouse AlexaFluor PLUS 555 and donkey anti-rabbit AlexaFluor PLUS 488 (Invitrogen)] and DAPI (Sigma) incubation for 15 mi. Slides were coverslipped with a fluorescence mounting medium (Agilent Technologies, Santa Clara, CA, United States). Imaging was performed on an Axio Z1 slide scanner (Zeiss) at 20x objective with Cyanine 3 (567 nm), FITC (475 nm), and DAPI (385 nm) fluorescent channels. Image acquisition and analysis were performed within ZEN software (ZEN 2.6 lite; Carl Zeiss). Annotation of specific functional structures seen in the H&E image from the library preparation slide was compared against the deeper consecutive multiplexed immunofluorescence image of the human cortical kidney tissue sections.

## Results

### Annotation of Cortical and Medullary Regions in Mouse ST-Seq Datasets

We used the pathologist's annotation of the functional mouse kidney regions (Figures 2B,C) to explore and predict functional nephron regions within the generated ST-seq dataset (38). Louvain clustering based on the K-nearest neighbor (KNN) of the ST-spots identified two to three distinct clusters in each sample (50). ST-spot clusters were then mapped to the H&E tissue images to examine the spatial distribution of the resulting clusters.

In female mice, three distinct clusters were mapped to the cortex and outer medulla, composed of the outer and inner stripe layers (Figures 2D,E). Within the cortex cluster, an additional small sub-cluster (Cluster 3 blue) was mapped to the edges of the tissue sections. This sub-cluster contained hemoglobin genes in the top 10 significant marker genes, implicating the presence of accumulated blood (Supplementary Table 3). Both spatial mapping and UMAP demonstrated colocalization of this sub-cluster with the cortex cluster (Cluster 0 yellow). Thus, we have classified them together as a cortex for further analysis.

In male mice, we noted two distinct clusters that mapped to the cortical and the outer stripe of the outer medulla (Figures 2D,E). Within the cortex cluster, an additional small sub-cluster (Cluster 2 green) was mapped to the edges of the outer stripe of the outer medulla. We observed that the top 10 significant genes within this sub-cluster contained genes that mapped to the female mice's outer stripe of the outer medulla (Supplementary Table 3). Both spatial mapping and UMAP demonstrated colocalization of this sub-cluster with the outer stripe of the outer medulla cluster (Cluster 1 orange). Therefore, we have classified them together as outer medulla for further analysis.

We observed that clusters mapped to the cortex contained marker genes for glomeruli (*Nphs2* and *Gpx3*; *p*_adj_ < 0.05). Clusters mapped to the outer stripe of the outer medulla contained marker genes for proximal tubules (*Acy3* and *Aqp1*; *p*_adj_ < 0.05). Clusters mapped to the inner stripe of the outer medulla contained marker genes for the loop of Henle (*Egf* and *Umod*; *p*_adj_ < 0.05) (51–53). Subsequent visualization of the clusters mapped to the H&E tissue images confirmed the presence of these dominant functional nephron structures in the mouse kidneys.

After implementing an unbiased clustering approach, we performed label transfer at the functional structure level to determine the cellular identities of all ST-spots (54, 55). The consensus annotations were then mapped to the H&E tissue images (Figures 2F,G). This consensus-based label transfer annotated the majority of the ST-spots in the cortex and the outer stripe of the outer medulla as proximal tubules (*Lrp2* and *Slc22a7*; *p*_adj_ < 0.05) and those in the inner stripe of the outer medulla as the loop of Henle (*Slc12a1* and *Umod*; *p*_adj_ < 0.05; Supplementary Table 4).

We performed deconvolution at the functional structure level in the mouse ST-seq datasets. This demonstrated that all the mouse ST-spots contained multiple functional structures (Figure 2H). Deconvolution within the ST-spots overlying the cortical regions detected a higher proportion of proximal tubule signatures and a lower proportion of glomerular signatures. Re-examination of the clusters mapped to the cortical region confirmed the expression of proximal tubule marker genes (51, 52).

### Annotation of Functional Structures Within the Human ST-Seq Datasets

We performed similar identification of functional structures, their transcriptional signatures, and spatial locations within the human cortical ST-seq datasets using Seurat clustering and label transfer (38). We initially defined the spatial organization of the human cortical kidney by performing Louvain clustering based on KNN to identify ST-spots with distinct transcriptome profiles. We mapped these cluster identities to the H&E tissue images (Figure 3B; Supplementary Figure 1B). For patient A, two clusters were mapped to the glomerular and mixed cortical renal parenchyma ST-spots. For patients B–D, three clusters were mapped to the glomerular, tubules, and mixed cortical renal parenchyma ST-spots. We observed that clusters mapping to the glomerular ST-spots contained marker genes for podocytes (*PODXL* and *NPHS2*; *p*_adj_ < 0.05; Supplementary Table 5) (51, 52). Clusters mapping to the tubules contained marker genes for proximal tubules (*LRP2* and *GPX3*; *p*_adj_ < 0.05) (51, 52). Concurrent assessment of the mapped clusters to the H&E tissue images revealed that glomeruli were the dominant functional nephron structures overlying the ST-spots.

We performed label transfer at functional structure level to determine the cellular identities of all ST-spots (Figure 3C; Supplementary Figure 1C) (6, 12). We found that the consensus-based label transfer resulted in the identification of collecting ducts (*AQP2* and *ATP6V0D2*; *p*_adj_ < 0.05), distal convoluted tubules (*SLC12A3* and *DEFB1*; *p*_adj_ < 0.05), glomeruli (*PODXL* and *NPHS2*; *p*_adj_ < 0.05), immune cells (*IL7R* and *CD86*; *p*_adj_ < 0.05), interstitium (*COL1A2* and *COL3A1*; *p*_adj_ < 0.05), loop of Henle (*UMOD* and *SLC12A1*; *p*_adj_ < 0.05), proximal tubules (*SLC22A8* and *ALDOB*; *p*_adj_ < 0.05) and vessels (*TAGLN, MYH11*, and *ELN*; *p*_adj_ < 0.05; Supplementary Table 6).

The consensus-based label transfer identified the primary functional structure within the cortical human kidney tissue as proximal tubules. We independently validated this result by comparing the cortical functional structures annotated by label transfer to the pathologist's annotation of the H&E images and multiplexed immunofluorescence (mIF) staining (Figure 3, Supplementary Figure 1). The label transfer, pathologist's H&E annotation, and mIF staining collectively identified glomeruli, vessels, and proximal tubules in the normal human cortical kidney tissues.

### Differential Expression Within Cortical Kidney Regions Between Species

**Figure 4:**
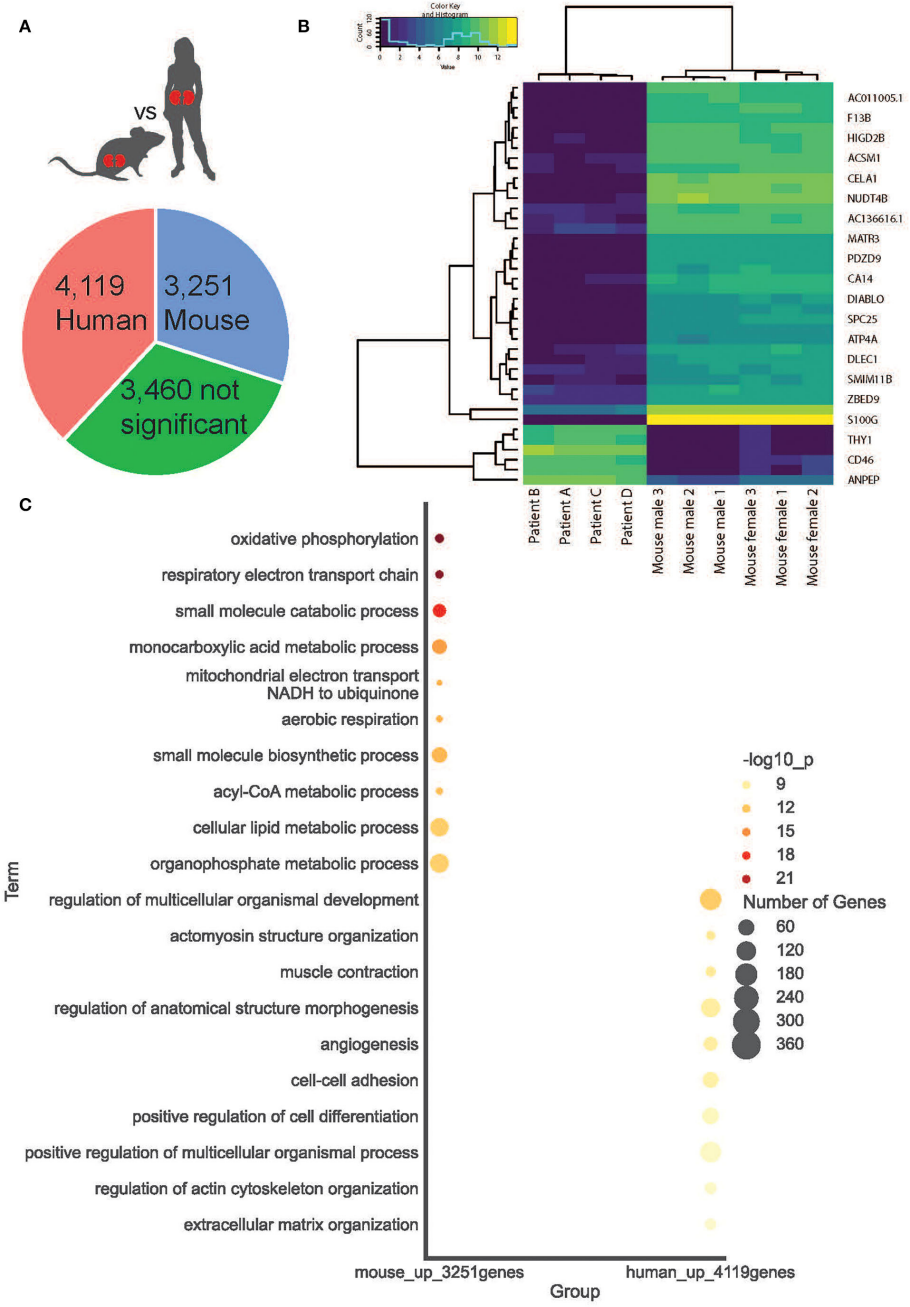
Cortical kidney genes differentially expressed between species. **(A)** A simple pie chart demonstrates the proportion of statistically significant DE genes identified within each species. **(B)** The top 20 DE genes between species are presented as a heat map. **(C)** Within mice and humans, the top 10 statistically significant Gene Ontology Biological Processes.

We compared gene signatures between human and mouse cortical kidney regions by identifying differentially expressed (DE) genes between the ST-seq datasets in humans and mice. Considering that the human ST-seq datasets comprised only cortical kidney, we used the pathologist's annotation to select the cortical kidney regions within the mouse ST-seq datasets. We identified 11,997 orthologous genes among the cortical kidney genes in the mouse ST-seq datasets (Supplementary Figure 6). After integration and removal of lowly expressed genes, 10,830 genes shared across the cortical kidney regions were used to test for DE genes and to assess functional and biological processes that vary between the species (Supplementary Table 7). In brief, we found 7,370 DE genes (FDR <0.05; no log-fold change cut-off) between human and mouse cortical kidney regions (Figure 4A). Examination of the top 20 DE genes showed high consistency across biological replicates and their distinct expression profiles between humans and mice (Figure 4B). The cortical location of the top 20 DE genes was further validated by their expression within cortical kidney cells in the Kidney Cell Explorer scRNA-seq database (https://cello.shinyapps.io/kidneycellexplorer/) and the Kidney Interactive Transcriptomics sn/scRNA-seq database (http://humphreyslab.com/SingleCell/, Supplementary Table 8) (56–58). We tested functional enrichment among all the significant DE genes, within Biological Processes Gene Ontology (GO:BP) terms (Figure 4C). We examined the top 20 GO:BP terms with the most significant *p*-values. In human cortical tissues, the most statistically significant GO:BP terms were associated with structural maintenance (Supplementary Table 9). In contrast, the most statistically significant GO:BP terms were associated with energy production and metabolic processes in mouse cortical regions (Supplementary Table 10).

### Cell-Cell Interaction Within and Between ST-Spots Containing Glomeruli in Human ST-Seq Datasets

**Figure 5:**
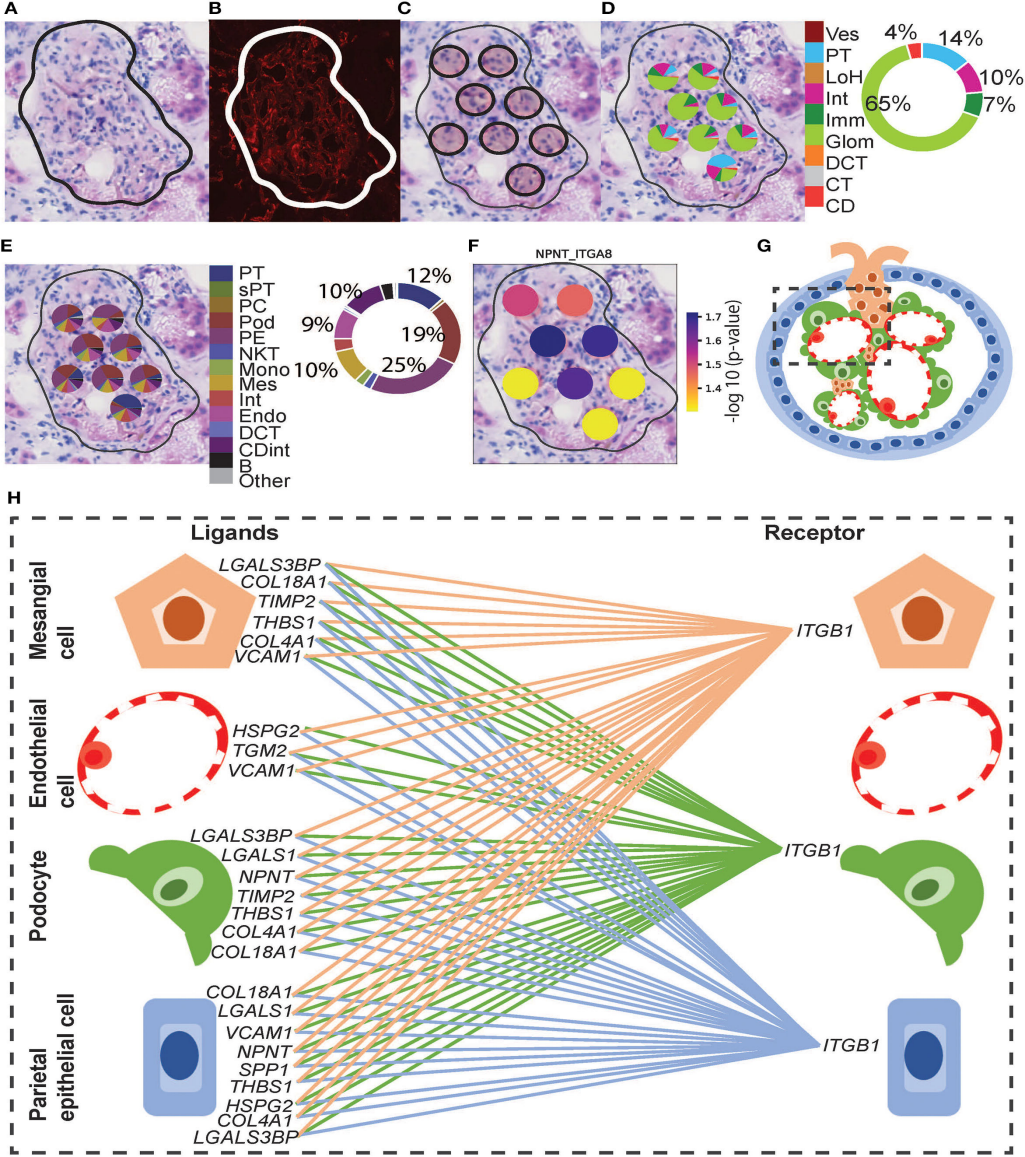
Integrative analysis of glomerular morphology, deconvolution, and cellular interactions in patient D. To confirm the morphology of glomerular functional structures, we investigated H&E and mIF images within a selected glomerulus. **(A)** Zoomed-in H&E image of the selected glomerulus annotated by the pathologist. **(B)** Anti-CD31 (red) immunofluorescence staining confirms the presence of endothelial cells and validates the pathologist's glomerular annotation. Next, we visualized the ST-spots underlying the glomerulus. **(C)** The positions of the eight underlying ST-spots were mapped within the selected glomerulus. To perform deconvolution, we selected the ST-spots identified by label transfer as glomeruli. **(D)** Deconvolution at the functional structure level for the selected glomerulus was mapped to the H&E image. The pie chart provides a summary of functional structures underlying all glomerular ST-spots in the entire tissue section for patient D. (**E)** Deconvolution at a cell-type level for the selected glomerulus was mapped to the H&E image. The pie chart provides a summary of the cell types underlying all glomerular ST-spots in the entire tissue section for patient D. Finally, we investigated our ST-seq datasets for cellular interactions in glomerular ST-spots. **(F)** The spatial expression of the *NPNT-ITGA8* L–R gene pair for the selected glomerulus was mapped to the H&E image. **(G)** A diagrammatic presentation of parietal epithelial, podocytes, endothelial, and mesangial cells that form the functional glomerular structures in mammalian cortical kidney regions. **(H)** The cellular interaction involved in extracellular matrix maintenance within the glomerulus for integrin receptor *ITGB1* was mapped between the glomerular cell types.

Functional structure level deconvolution results were used to select the ST-spots that contained glomerular structures (Figure 5). In these selected glomerular ST-spots, we further deconvoluted to cell-type level and found that podocytes, mesangial, endothelial, and parietal epithelial cells were the major cell types. We identified co-expression of 330 L–R gene pairs within and between glomerular ST-spots (Supplementary Table 11). We selected the top 40 L–R gene pairs identified as the most statistically significant (*p*_adj_ <0.05) within and between glomerular ST-spots (Table 2) (57–61). We identified 23 L–R gene pairs involving integrin receptors *ITGA3, ITGAV, ITGA8, ITGB1, ITGB5*, and laminin receptor *RPSA* within the extracellular matrix maintenance group. We identified five L–R gene pairs with co-expression of vascular endothelial growth factor *VEGF-A, KDR*, and *FLT1* within the angiogenic regulation group. Additional novel L–R gene pairs *FGF-NRP1, THBS1-SDC4*, and *ANXA2-ROBO4* are non-*VEGF* L–R pairs, identified within the angiogenic regulation group and previously shown to regulate and maintain the microvasculature within glomeruli (62–65). We identified six L–R gene pairs with co-expression of Human Leukocyte Antigens (*HLA-A, HLA-B, and HLA-F*) ligands, Amyloid beta Precursor Protein (*APP*), Macrophage migration Inhibitory Factor (*MIF*), and Megalin (*LRP2*) within the immune and endocytic activity group. Additional novel L–R gene pairs *GRN-SORT1* and *TIMP1-CD63* identified within the immune and endocytic activity group are known L–R pairs within the nervous system but novel within the glomerular structure (66–68).

**Table 2:** CCI identified within and between glomerular ST-spots.

** 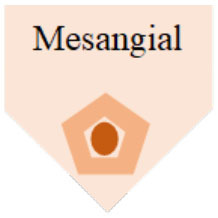 **	** 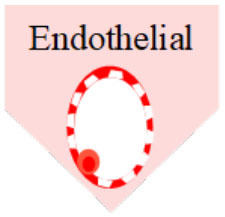 **	** 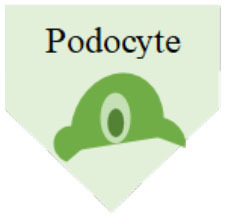 **	** 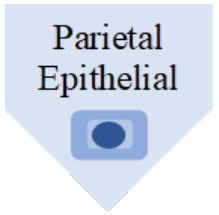 **	
Extracellular matrix maintenance				
*CALR- ITGA3*	*CALR- ITGA3*	*CALR- ITGA3*	*CALR- ITGA3*	(69–71)
*TIMP2-ITGA3*	*TIMP2-ITGA3*	*TIMP2-ITGA3*	*TIMP2-ITGA3*	(72–74)
*THBS1-ITGA3*	*THBS1-ITGA3*	*THBS1-ITGA3*	*THBS1-ITGA3*	(75–77)
*SPP1-ITGAV*	*SPP1-ITGAV*	*SPP1-ITGAV*	*SPP1-ITGAV*	(78)
*CALR- ITGAV*	*CALR- ITGAV*	*CALR- ITGAV*	*CALR- ITGAV*	(71, 79)
*CX3CL1- ITGAV*	*CX3CL1ITGAV*	*CX3CL1- ITGAV*	*CX3CL1- ITGAV*	(80–82)
*COL4A1-ITGAV*	*COL4A1-ITGAV*	*COL4A1- ITGAV*	*COL4A1- ITGAV*	(83, 84)
*COL4A3- ITGAV*	*COL4A3- ITGAV*	*COL4A3- ITGAV*	*COL4A3- ITGAV*	(85, 86)
*NPNT- ITGA8*	*NPNT- ITGA8*	*NPNT- ITGA8*	*NPNT- ITGA8*	(79, 87–90)
*SPP1-ITGB1*	*SPP1-ITGB1*	*SPP1-ITGB1*	*SPP1-ITGB1*	(79, 91–95)
*TIMP2- ITGB1*	*TIMP2- ITGB1*	*TIMP2- ITGB1*	*TIMP2- ITGB1*	(73, 74)
*NPNT- ITGB1*	*NPNT- ITGB1*	*NPNT- ITGB1*	*NPNT- ITGB1*	(79, 90, 96, 97)
*COL18A1- ITGB1*	*COL18A1- ITGB1*	*COL18A1- ITGB1*	*COL18A1- ITGB1*	(98–100)
*LGALS1- ITGB1*	*LGALS1- ITGB1*	*LGALS1- ITGB1*	*LGALS1- ITGB1*	(101, 102)
*THBS1- ITGB1*	*THBS1- ITGB1*	*THBS1- ITGB1*	*THBS1- ITGB1*	(103, 104)
*LGALS3BP- ITGB1*	*LGALS3BP- ITGB1*	*LGALS3BP-ITGB1*	*LGALS3BP- ITGB1*	(102, 105)
*COL4A1-ITGB1*	*COL4A1-ITGB1*	*COL4A1-ITGB1*	*COL4A1-ITGB1*	(86, 106)
*HSPG2-ITGB1*	*HSPG2-ITGB1*	*HSPG2-ITGB1*	*HSPG2-ITGB1*	(107–109)
*TGM2- ITGB1*	*TGM2- ITGB1*	*TGM2- ITGB1*	*TGM2- ITGB1*	(110, 111)
*VCAM1- ITGB1*	*VCAM1- ITGB1*	*VCAM1- ITGB1*	*VCAM1- ITGB1*	(112–114)
*SPP1- ITGB5*	*SPP1- ITGB5*	*SPP1- ITGB5*	*SPP1- ITGB5*	(115, 116)
*THY1- ITGAV*	*THY1- ITGAV*	*THY1- ITGAV*	*THY1- ITGAV*	(117, 118)
*LAMB2- RPSA*	*LAMB2- RPSA*	*LAMB2- RPSA*	*LAMB2- RPSA*	(119, 120)
Angiogenic regulation				
*TIMP3-KDR*	*TIMP3-KDR*	*TIMP3-KDR*	*TIMP3-KDR*	(121–123)
*VEGF-A-FLT1*	*VEGF-A-FLT1*	*VEGF-A-FLT1*	*VEGF-A-FLT1*	(121, 124–126)
*VEGF-A-NRP1*	*VEGF-A-NRP1*	*VEGF-A-NRP1*	*VEGF-A-NRP1*	(121, 125, 127, 128)
*VEGF-A-KDR*	*VEGF-A-KDR*	*VEGF-A-KDR*	*VEGF-A-KDR*	(121, 125, 126, 129)
*COL18A1-KDR*	*COL18A1-KDR*	*COL18A1-KDR*	*COL18A1-KDR*	(121, 130)
*FGF1-NRP1*	*FGF1-NRP1*	*FGF1-NRP1*	*FGF1-NRP1*	(128)
*THBS1-SDC4*	*THBS1-SDC4*	*THBS1-SDC4*	*THBS1-SDC4*	(63, 64, 131)
*ANXA2-ROBO4*	*ANXA2-ROBO4*	*ANXA2-ROBO4*	*ANXA2-ROBO4*	(65, 132)
Immune and endocytic activity				
*APP-CD74*	*APP-CD74*	*APP-CD74*	*APP-CD74*	(133–137)
*APP-NCSTN*	*APP-NCSTN*	*APP-NCSTN*	*APP-NCSTN*	(136, 138, 139)
*MIF-CD74*	*MIF-CD74*	*MIF-CD74*	*MIF-CD74*	(140, 141)
*HLA-A-APLP2*	*HLA-A-APLP2*	*HLA-A-APLP2*	*HLA-A-APLP2*	(136, 142–144)
*HLA-B-CANX*	*HLA-B-CANX*	*HLA-B-CANX*	*HLA-B-CANX*	(144–146)
*HLA-F-B2M*	*HLA-F-B2M*	*HLA-F-B2M*	*HLA-F-B2M*	(144, 147)
*GRN-SORT1*	*GRN-SORT1*	*GRN-SORT1*	*GRN-SORT1*	(68, 148, 149)
*TIMP1-CD63*	*TIMP1-CD63*	*TIMP1-CD63*	*TIMP1-CD63*	(150–152)
*APOE-LRP2*	*APOE-LRP2*	*APOE-LRP2*	*APOE-LRP2*	(153–155)

*Key, L-R presence indicated by back text and absence indicated by the gray text*.

*The literature-based locations of the top 40 glomerular L–R gene pairs were confirmed in sc/snRNA-seq datasets, as an expression within glomerular cell types. In particular the sc/snRNA-seq datasets within the Kidney Interactive Transcriptomics [Healthy Adult Kidney - Epithilia (57), Healthy Mouse Dataset (58), Human Diabetic Kidney (59), and Human Kidney snRNA/ATAC-seq (60), and The Human Nephrogenesis Atlas (Human week 14 scRNA-seq) (61) were utilized*.

### CCI Within and Between ST-Spots Containing Proximal Tubules–Peritubular Capillaries in Human ST-Seq Datasets

**Figure 6:**
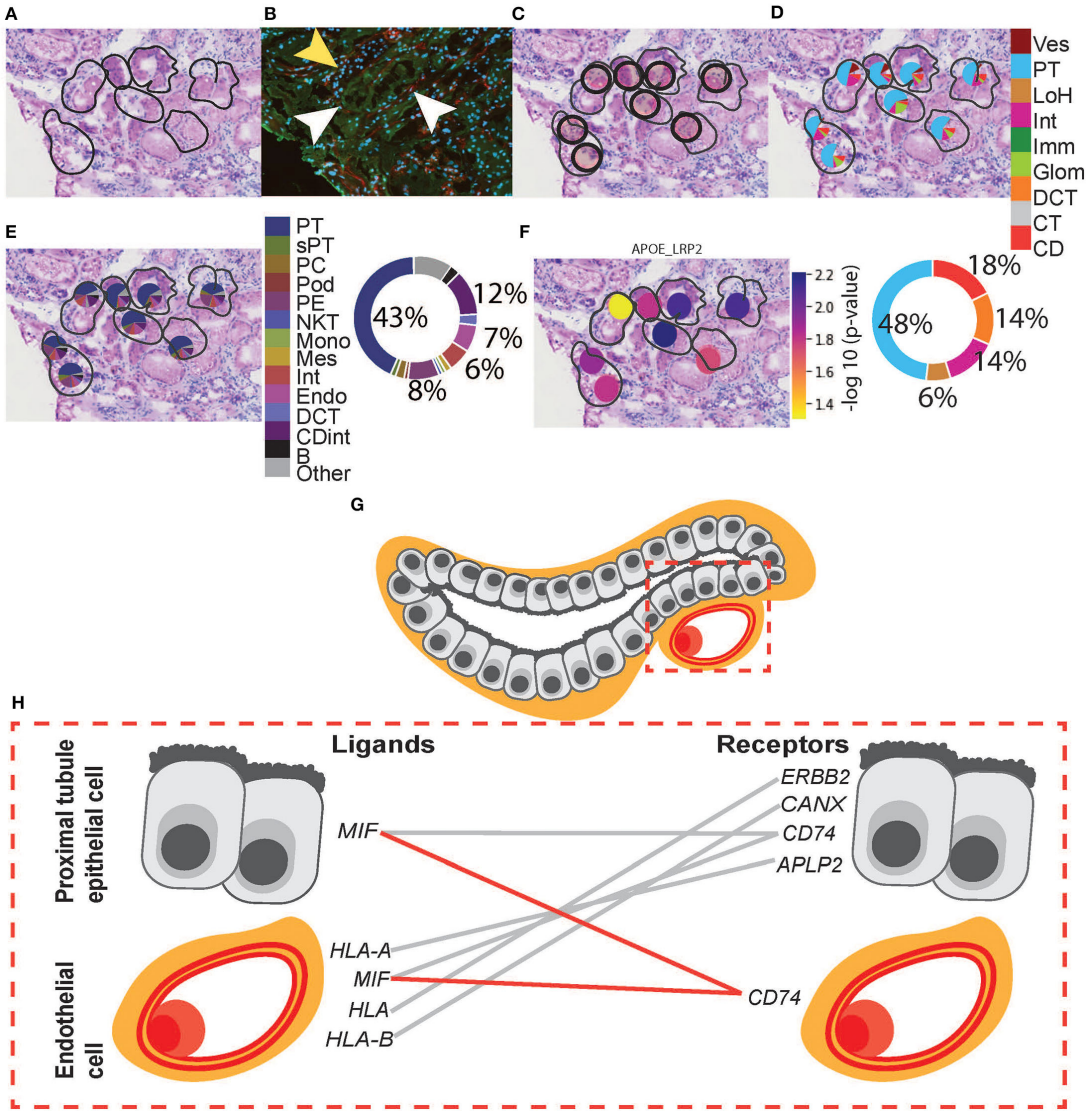
Integrative analysis of PT–PC morphology, deconvolution, and cellular interactions in patient D. To confirm the morphology of PT, we investigated H&E and mIF images within an ROI. **(A)** Zoomed-in H&E image of PT was annotated by the pathologist. **(B)** mIF staining with anti-AQP1 (green) demonstrates proximal tubules, anti-CD31 (red; white arrowheads) demonstrates peritubular capillaries, and DAPI (blue) demonstrates nuclei. The mIF staining confirms the presence of PT and PC structures and validates the pathologist's annotation. (Note: yellow arrowhead denotes tubular patterned DAPI staining with an absence of anti-AQP1 indicating the presence of non-PT structures). **(C)** The positions of eight underlying ST-spots were mapped within the selected ROI. To perform deconvolution, we selected the ST-spots identified by label transfer as PT. **(D)** Deconvolution at the functional structure level for the selected eight PT ST-spots was mapped to the H&E image. The pie chart provides a summary of functional structures underlying all PT ST-spots in the entire tissue section for patient D. **(E)** Deconvolution at the cell-type level for the selected eight PT ST-spots was mapped to the H&E image. The pie chart provides a summary of the cell types underlying all PT ST-spots in the entire tissue section for patient D. Finally, we investigated our ST-seq datasets for cellular interactions in PT–PC ST-spots. **(F)** The spatial expression of the *APOE-LRP2* L–R gene pair for the selected PT was mapped to the H&E image. **(G)** A diagrammatic presentation of PT epithelial cells and PC endothelial cells within mammalian cortical kidney regions. **(H)** The cellular interactions involved in immune modulation within PT–PC cells were mapped. Functional structure level key: Ves—vessels, PT—proximal tubules, LoH—loop of Henle, Int—interstitium, Imm—immune cells, Glom—glomeruli, DCT—distal convoluted tubule, CT—connecting tubule, and CD—collecting duct. Cell-type level key: PT—proximal tubule cell, sPT—proximal straight tubule cell, PC—principal cell, Pod—podocytes, PE—parietal epithelial cell, NKT—natural killer T-cell, Mono—monocytes, Mes—mesangial cell, Int—interstitium, Endo—endothelial cell, DCT —distal convoluted tubule cell, CDint - collecting duct intercalated cell and B—B cell.

We extended the CCI investigation to ST-spots containing PT–PC to investigate potential cross-talk within and between these cell types. To perform this analysis, we selected human ST-spots that after deconvolution was annotated to contain proximal tubule cells plus endothelial cells, but not annotated as glomerular endothelial cell types (Figure 6). Again, we tested the >2,000 L–R pairs curated in the connectomeDB2020 database, using stLearn CCI analysis with both within and between spots (48). We identified significant co-expression of 170 L–R gene pairs in PT–PC ST-spots (Supplementary Table 11). We selected the top 20 L–R gene pairs identified as statistically significant (*p*_adj_ <0.05) within and between PT–PC ST-spots (Table 3) (57–61). We identified six L–R gene pairs with co-expression of *LRP2, APP*, Low-Density Lipoprotein Receptor (*LDLR*), and TIMP Metallopeptidase Inhibitor 1 (*TIMP1*) within the transportation and signaling group. We identified eight L—R gene pairs with co-expression of Integrin (*ITGB1, ITGB5*, and *ITGAV*), CD44 molecule, and Epithelial Cell Adhesion Molecule (*EPCAM*) within the adhesion group. We identified four L–R gene pairs with the co-expression of *HLA* and *MIF* within the immune modulation group. Finally, within the angiogenic regulation group, we identified two L–R gene pairs with the co-expression of Thrombospondin 1 (*THBS1*) and Syndecan (*SDC1* and *SDC4*).

**Table 3:** CCI identified within and between proximal tubule and endothelial ST-spots.

** 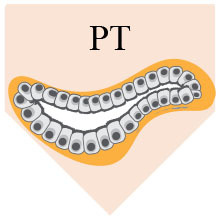 **	** 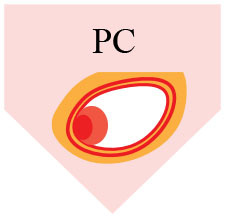 **	
Transportation and signaling		
*APOE-LRP2*	*APOE-LRP2*	(156)
*APP-CD74*	*APP-CD74*	(133)
*ALB-LRP2*	*ALB-LRP2*	(157)
*APOE-LDLR*	*APOE-LDLR*	(158)
*SERPINE1-LRP2*	*SERPINE1-LRP2*	(159)
*TIMP1-CD63*	*TIMP1-CD63*	(160, 161)
Adhesion		
*THBS1-ITGB1*	*THBS1-ITGB1*	(162, 163)
*COL18A1-ITGB1*	*COL18A1-ITGB1*	(130, 164, 165)
*PLG-ITGB1*	*PLG-ITGB1*	(166)
*SPP1-ITGB5*	*SPP1-ITGB5*	(167)
*SPP1-ITGB1*	*SPP1-ITGB1*	(94, 168)
*SPP1-ITGAV*	*SPP1-ITGAV*	(92, 169)
*SPP1-CD44*	*SPP1-CD44*	(170)
*EPCAM- EPCAM*	*EPCAM- EPCAM*	(171)
Immune modulation		
*HLA-B-CANX*	*HLA-B-CANX*	(146, 172)
*HLA-A-APLP2*	*HLA-A-APLP2*	(143, 173, 174)
*MIF-CD74*	*MIF-CD74*	(175)
*HLA-ERBB2*	*HLA-ERBB2*	(163)
Angiogenic regulation		
*THBS1-SDC1*	*THBS1-SDC1*	(64)
*THBS1-SDC4*	*THBS1-SDC4*	(64, 176, 177)

*The literature based location of the top 20 PT-PC L–R gene pairs were confirmed in sc/snRNA-seq datasets, as an expression within proximal tubule (segments 1, 2, and 3), peritubular capillary, and endothelial cell types. In particular, the sc/snRNA-seq datasets within the Kidney Interactive Transcriptomics (Healthy Adult Kidney—Epithilia (57), Healthy Mouse Dataset (58), Human Diabetic Kidney (59) and Human Kidney snRNA/ATAC-seq (60), and The Human Nephrogenesis Atlas [Human week 14 scRNA-seq (61)] were utilized*.

## Discussion

Available transcriptome profiles of normal nephrons have utilized bulk and/or scRNA-seq/snRNA-seq methods requiring the manipulation of tissue, including tissue homogenization or cell dissociation, resulting in the loss of crucial spatial information. In this study, we performed ST-seq to resolve gene expression within intact normal tissues of six mice and four human kidneys. We captured more genes and reads in the mouse kidneys (median genes 3,310–5,994 and median reads 10,491–31,145) compared to human kidneys (median genes 674–1,519 and median reads 1,139–3,037). Within the captured ST-seq datasets, we defined the spatial location of specific nephron segments, compared DE genes between species, and spatially mapped the putative cellular communication occurring in glomerular and PT–PC regions in the human ST-seq datasets.

In the mouse ST-seq datasets, we defined the functional regions with KNN clustering to the cortex and the outer and inner stripes of the outer medulla regions. We confirmed the cluster identities by marker gene expression and found a direct correlation with the pathologist's annotation. However, label transfer–based annotation of the functional nephron regions using publicly available mouse scRNA-seq datasets identified only two distinct clusters (54, 55). The outer stripe of the outer medulla was indistinguishable from the cortical layer in female and male mice kidneys. We attributed this curious result to the large ST-spot size and the small size and dense assembly of cortical functional structures, such as the proximal tubules, in mouse kidneys. To address the latter, we performed deconvolution with the mouse ST-seq datasets and found multiple functional structures within all 100 μm ST-spots. Furthermore, deconvolution within both the cortex and the outer stripe of the outer medulla identified a higher proportion of proximal tubule signatures—a stochastic variation noted by other transcriptome studies (51, 178, 179). Therefore, we conclude that the discrepancy between cluster and pathologist annotation against the label transfer annotations occurred due to the dense assembly of functional structures in mouse kidneys, resulting in the capture of multiple structures in individual ST-spots.

In the human ST-seq datasets, we defined glomerular, collecting duct, and mixed cortical renal parenchyma ST-spots with KNN clustering. However, distinct functional nephron tubular segments were not apparent by clustering. We, therefore, performed further label transfer–based annotation of functional structures using published human kidney snRNA-seq and scRNA-seq datasets as references (6, 12). This resulted in the annotation of collecting ducts, distal convoluted tubules, glomeruli, immune cells, interstitium, the loop of Henle, proximal tubules, and vessels. The low immune infiltrate within the normal human cortical kidney tissue has been attributed to normal immune-surveillance/immune-regulatory functions (12, 51, 60, 179–189). We checked the cluster identities and label transfer annotations against marker gene expression, the pathologist's annotation, and mIF staining, demonstrating consistent agreement of the major functional nephron structures in normal human cortical kidney tissue.

We subsequently performed DE gene analysis between mouse and human cortical kidney regions. In this study, 7,370 DE genes (*p* <0.05) were identified between mouse and human cortical kidney regions and were tested for functions associated with the GO:BP terms. The top 10 statistically significant GO:BP terms up-regulated within the mouse cortical regions compared to humans associated with energy production and metabolic processes. This higher metabolic rate is a known phenomenon in mouse tissue, however, the actual cause remains unknown (190, 191). We hypothesize that some of the interspecies variations between our normal mice and human kidneys may be due to differences in age and environment (192–198). The mice in our study were 8 weeks old corresponding to humans <20 years of age and the human samples were from patients in their fifth decade of life. Therefore, the changes to mitochondrial energy production and metabolic processes detected between species may be secondary to the large differences in relative age and environment.

In the human ST-seq datasets, we investigated CCI in glomerular and PT–PC ST-spots, using L–R gene co-expression. In the glomerular ST-spots, we identified co-expression of 300 L–R gene pairs but focused on the top 40 L–R gene pairs (*p*_adj_ < 0.05). Consistent with published sc/snRNA-seq datasets (57–60), these top 40 L–R pairs were associated with structural, vascular, and/or immune interactions within and between mesangial, endothelial, podocytes, and parietal epithelial cells. The glomeruli are unique functional filtration structures composed of tufts of vascular endothelial capillaries surrounded by mesangial, podocyte, and parietal epithelial cells (3, 199). The mesangial cells, podocytes, and endothelial cells secrete extracellular matrix (ECM) components to establish a glomerular basement membrane (GBM) and form the glomerular filtration barrier, which allows fluid and solutes to pass into the nephron (200). ECM components such as integrins facilitate important signaling interactions between the mesangial cells, podocytes, and endothelial cells that surround and maintain the GBM (121, 201). Integrins are a large family of transmembrane receptors which, upon ligand activation, control signal transduction, cell adhesion, proliferation, and ECM maintenance (91, 200, 202–204). Consistent with expectations, 22 out of the top 40 L–R gene pairs identified were involved with integrin receptors *ITGA3, ITGAV, ITGA8, ITGB1*, and *ITGB5*. Moreover, five L–R gene pairs were involved in the regulation of angiogenesis and glomerular filtration barrier maintenance *via* VEGF-mediated signaling.

In the PT–PC ST-spots, we identified co-expression of 170 L–R gene pairs but focused on the top 20 L–R gene pairs (*p*_adj_ < 0.05). Consistent with published sc/snRNA-seq datasets (57–60), these top 20 L–R pairs were associated with lipid and protein transportation and signaling, adhesion, and/or immune interactions within and between proximal tubule epithelial cells and peri-tubular capillary endothelial cells. Proximal tubules are primarily responsible for the reabsorption of amino acids, glucose, solutes, and low–molecular weight proteins from the glomerular filtrate (205). Components reabsorbed from the filtrate are then taken up into the bloodstream *via* peritubular capillaries surrounding the proximal tubules. Consistent with expectations, six L–R gene pairs identified were involved in transportation and signaling facilitated by proximal tubule-specific endocytic receptors *LRP2* and *APP*. Eight L–R gene pairs identified were involved in cell adhesion primarily involving integrin-based interactions between proximal tubule cells aside from a predicted tubulo-vascular interaction involving *COL18A1-ITGB1*. Four L–R gene pairs identified were linked to immune modulation *via* the formation of the MHC class I loading complex *HLA* and *MIF*. Furthermore, two L–R gene pairs identified were linked to vascular maintenance *via SDC1* and *SDC4*. The identified top L–R gene pairs within and between glomerular and PT–PC ST-spots were validated by both localization and co-expression within the required cell types in published sc/snRNA-seq datasets (57–60). Additional identification of pathways established as fundamental to normal kidney function in published literature act as further validation of the specificity of the ST-seq approach for examining CCI within the glomerular and tubular compartments.

Our generated ST-seq datasets and analysis provide demonstration and confirmation of normal kidney tissue and physiological pathways. This is anticipated to assist with the future description and understanding of molecular signals and pathways in states of kidney disease, and thus support the development of therapeutics and diagnostic interventions for clinical translation.

## Data Availability Statement

The datasets presented in this study can be found in online repositories. The names of the repository/repositories and accession number(s) can be found below:

− The human and mouse kidney ST-seq datasets and codes are publicly available here: GitHub, https://github.com/BiomedicalMachineLearning/SpatialKidney.

− The raw data are publicly available here: ArrayExpress, https://www.ebi.ac.uk/arrayexpress, E-MTAB-11721.

## Ethics Statement

The studies involving human participants were reviewed and approved by Royal Brisbane and Women's Hospital Human Research Ethics Committee (Reference Number 2002/011). The patients/participants provided their written informed consent to participate in this study. The animal study was reviewed and approved by University of Queensland Animal Ethics Committee (UQDI/452/16 and IMB123/18).

## Author Contributions

AR, AC, AK, HH, QN, and AM conceived and designed the study. AR, PL, SY, ST, JC, and SA carried out the experiments. AR, MN, AK, HH, and AM reviewed the patient data. DP, XT, LG, and QN performed the bioinformatics analyses. AR, AK, AS, and LF performed the histological examination of the kidney. AR, DP, XT, NM, LG, AK, HH, AC, QN, and AM drafted the article. All authors revised and approved the final version of the manuscript. All authors contributed to the article and approved the submitted version.

## Funding

This study was supported by funding from Pathology Queensland-Study, Education and Research Committee, Royal Brisbane and Women's Hospital Foundation Project Grant 2019, Robert and Janelle Bird Postdoctoral Research Fellowship 2020, and the University of Queensland (UQ)-Genome Innovation Hub. AR is supported by an Australian Government Research Training Program (RTP) Scholarship. AM is supported by a Queensland Health Advancing Clinical Research Fellowship.

## Conflict of Interest

The authors declare that the research was conducted in the absence of any commercial or financial relationships that could be construed as a potential conflict of interest.

## Publisher's Note

All claims expressed in this article are solely those of the authors and do not necessarily represent those of their affiliated organizations, or those of the publisher, the editors and the reviewers. Any product that may be evaluated in this article, or claim that may be made by its manufacturer, is not guaranteed or endorsed by the publisher.
