# Supplementary material for: Spatially Resolved Transcriptomes of Mammalian Kidneys Illustrate the Molecular Complexity and Interactions of Functional Nephron Segments
Source: Front Med (Lausanne). 2022 Jul 7;9:873923. doi: 10.3389/fmed.2022.873923 (PMC9300864; doi:10.3389/fmed.2022.873923)

Supplementary Figure 1

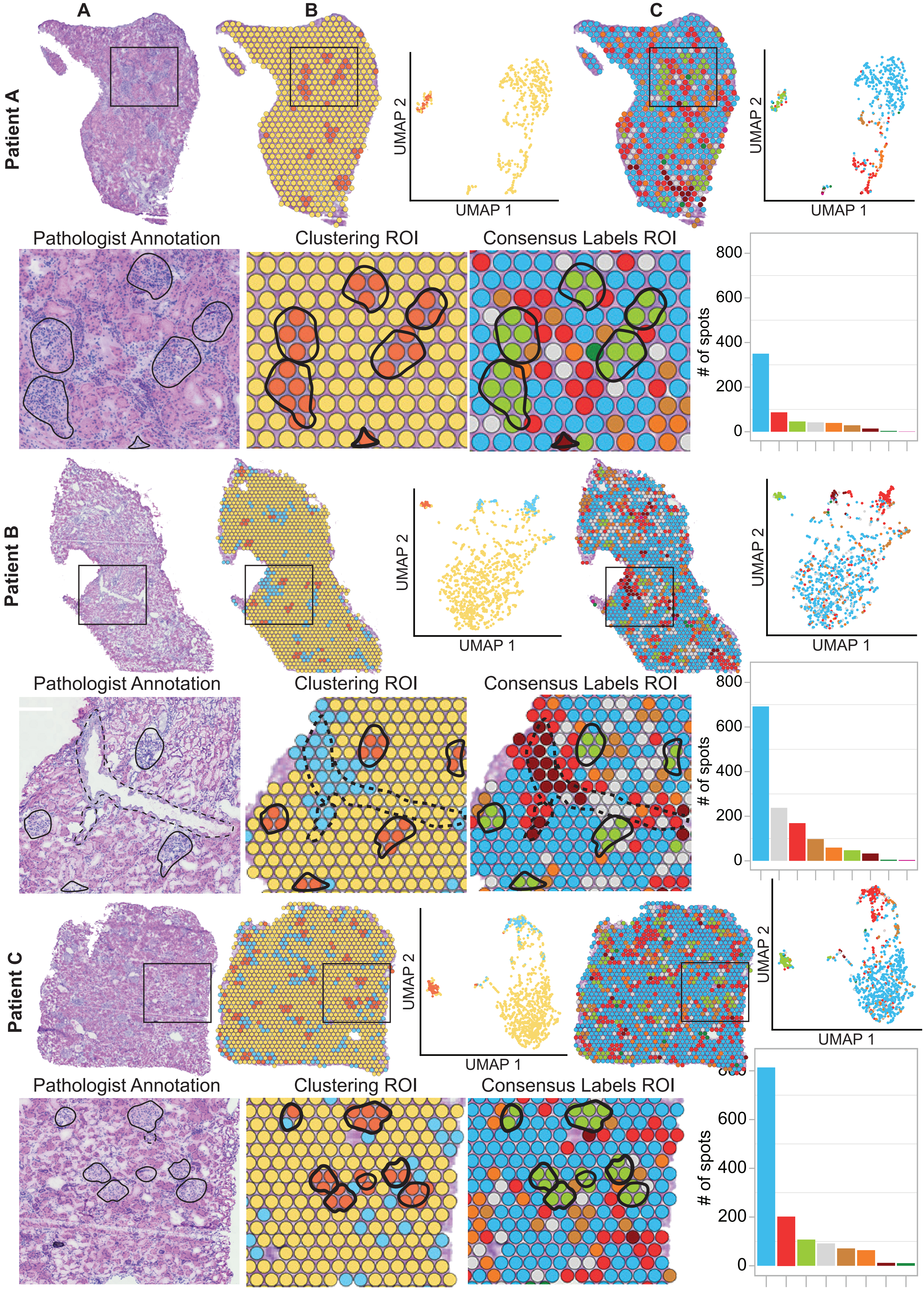

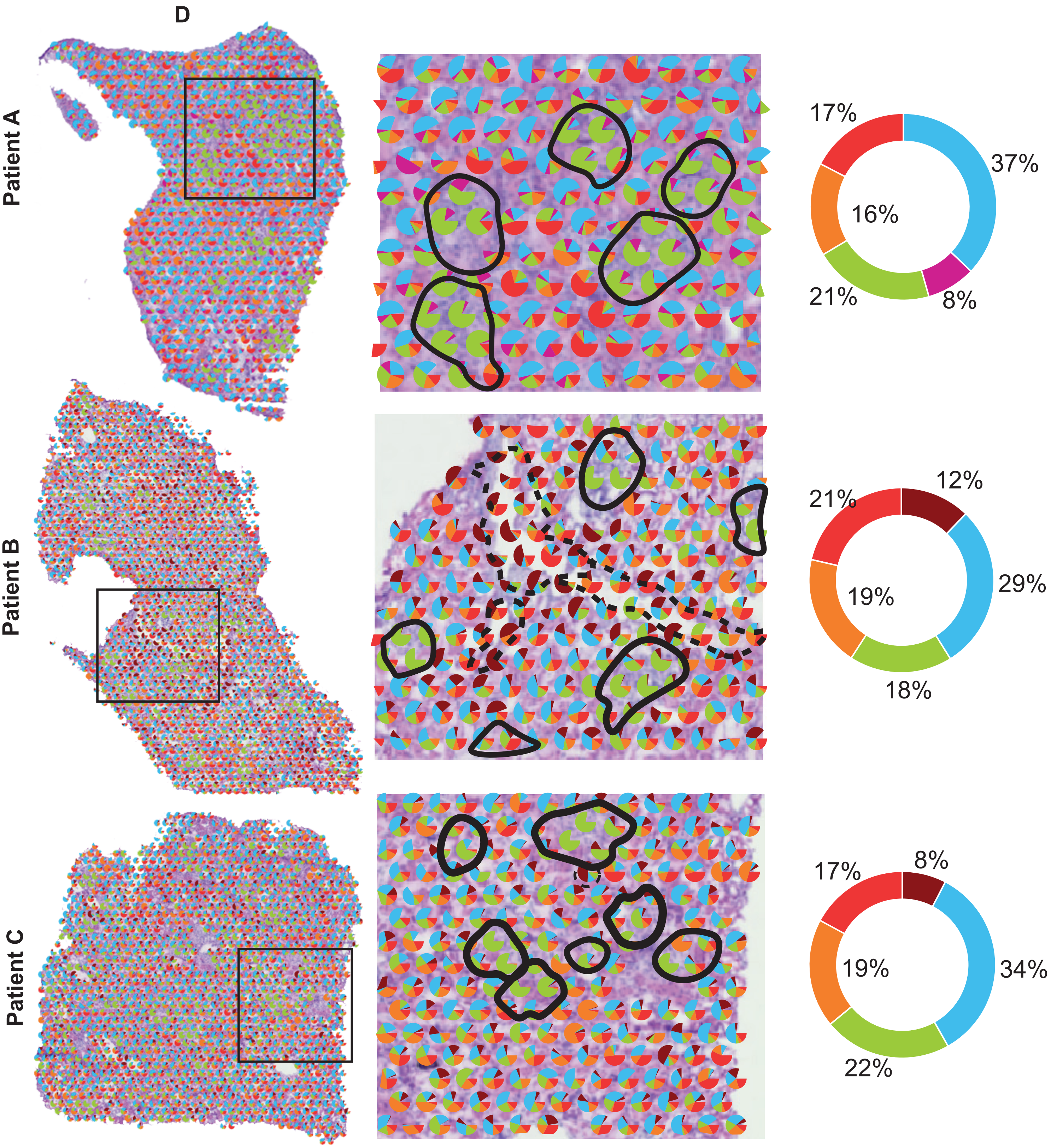
D
Patient A
Patient B
Patient C
17%
37%
16%
21%
8%
21%
12%
29%
19%
18%
17%
8%
34%
19%
22%

Supplementary Figure 2

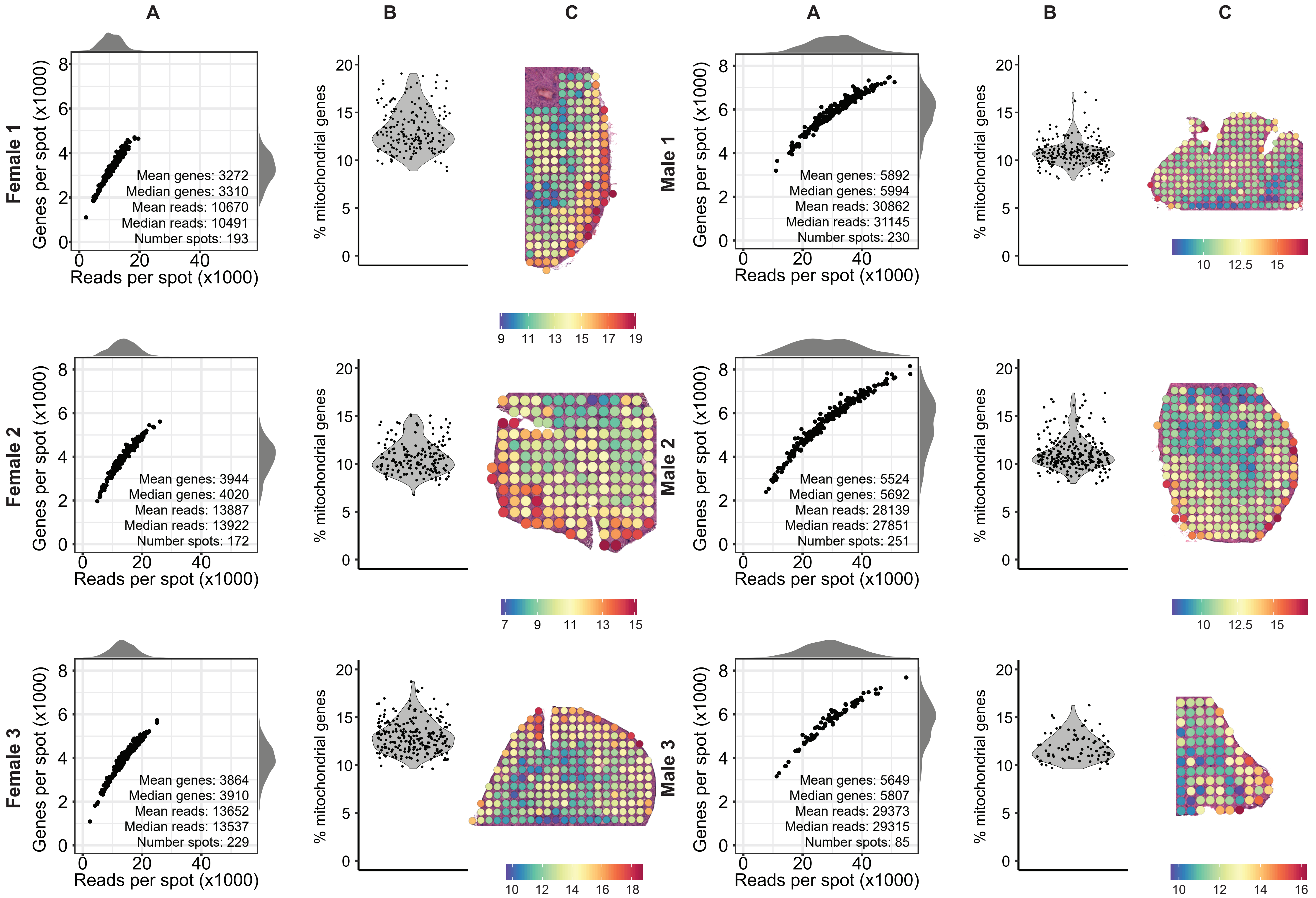

Supplementary Figure 3

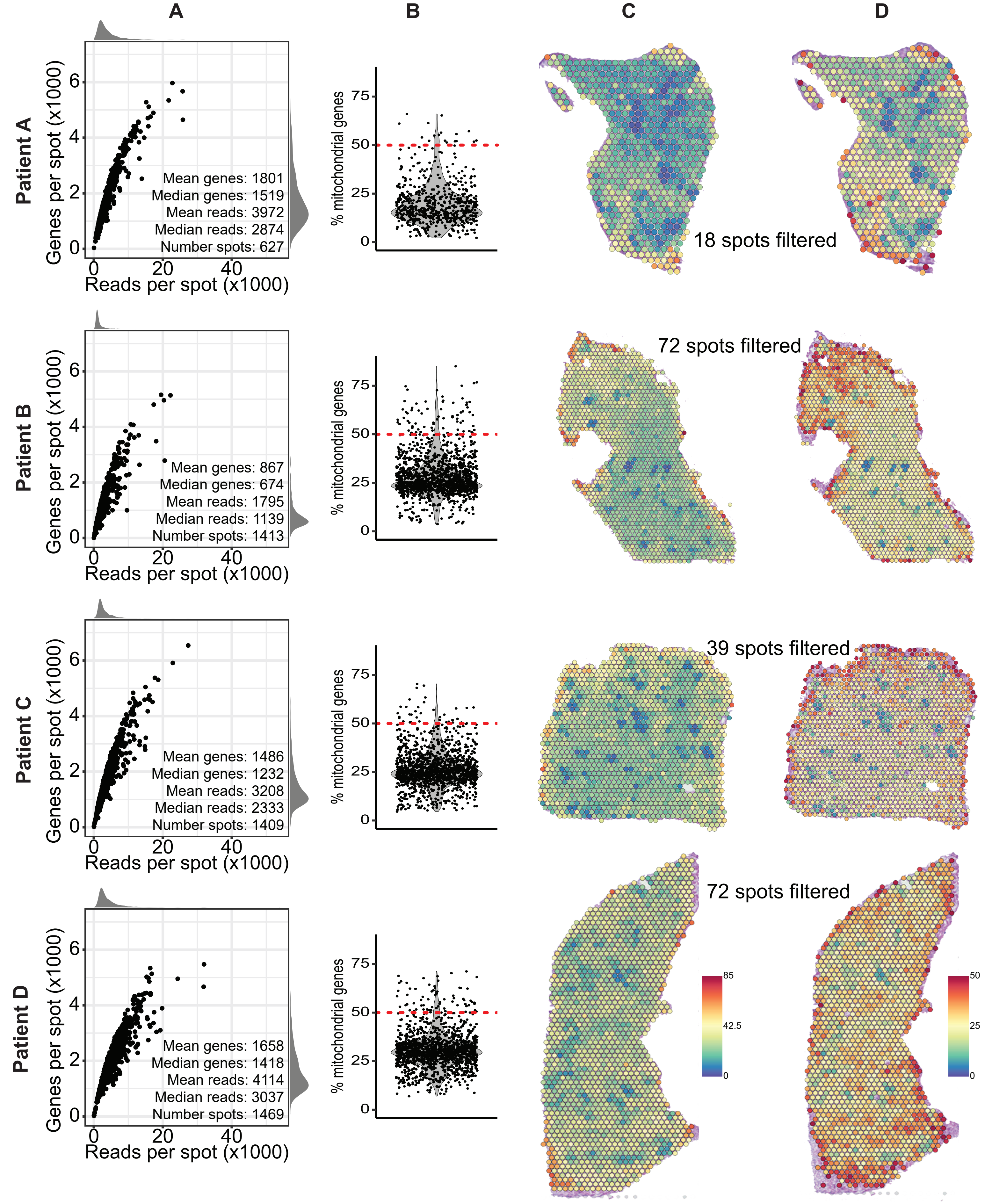

Supplementary Figure 4

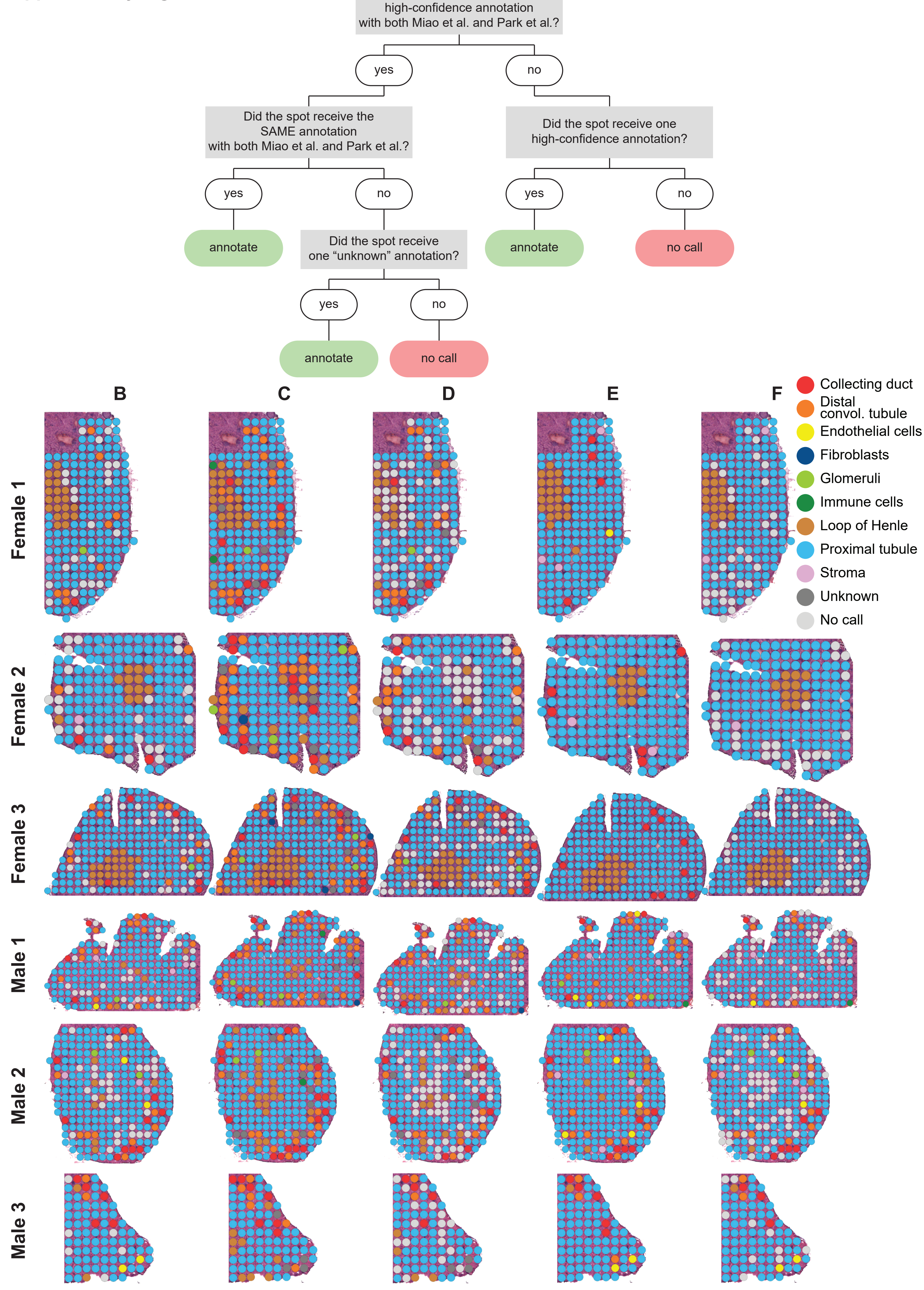

Supplementary Figure 5

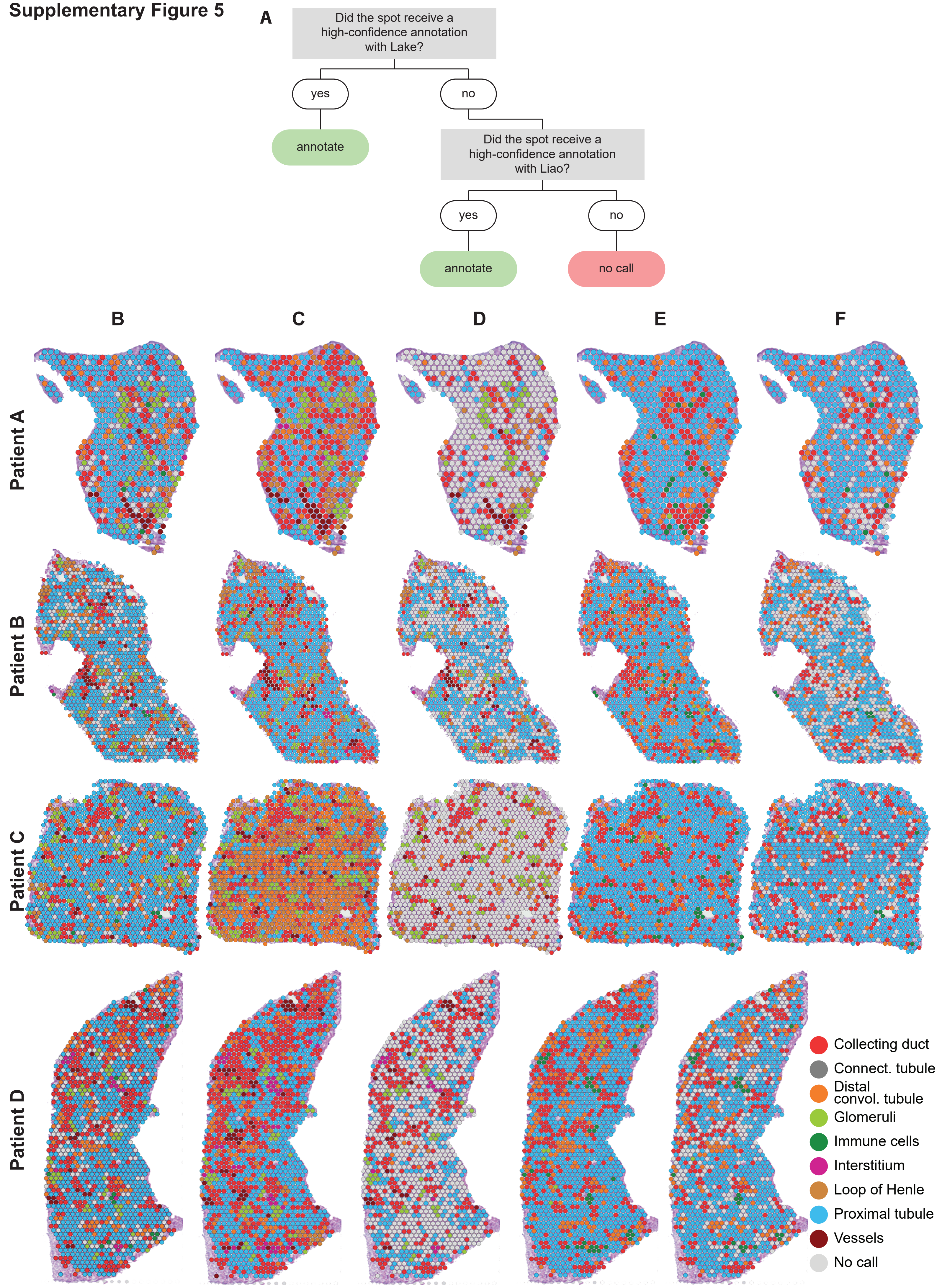

Supplementary Figure 6

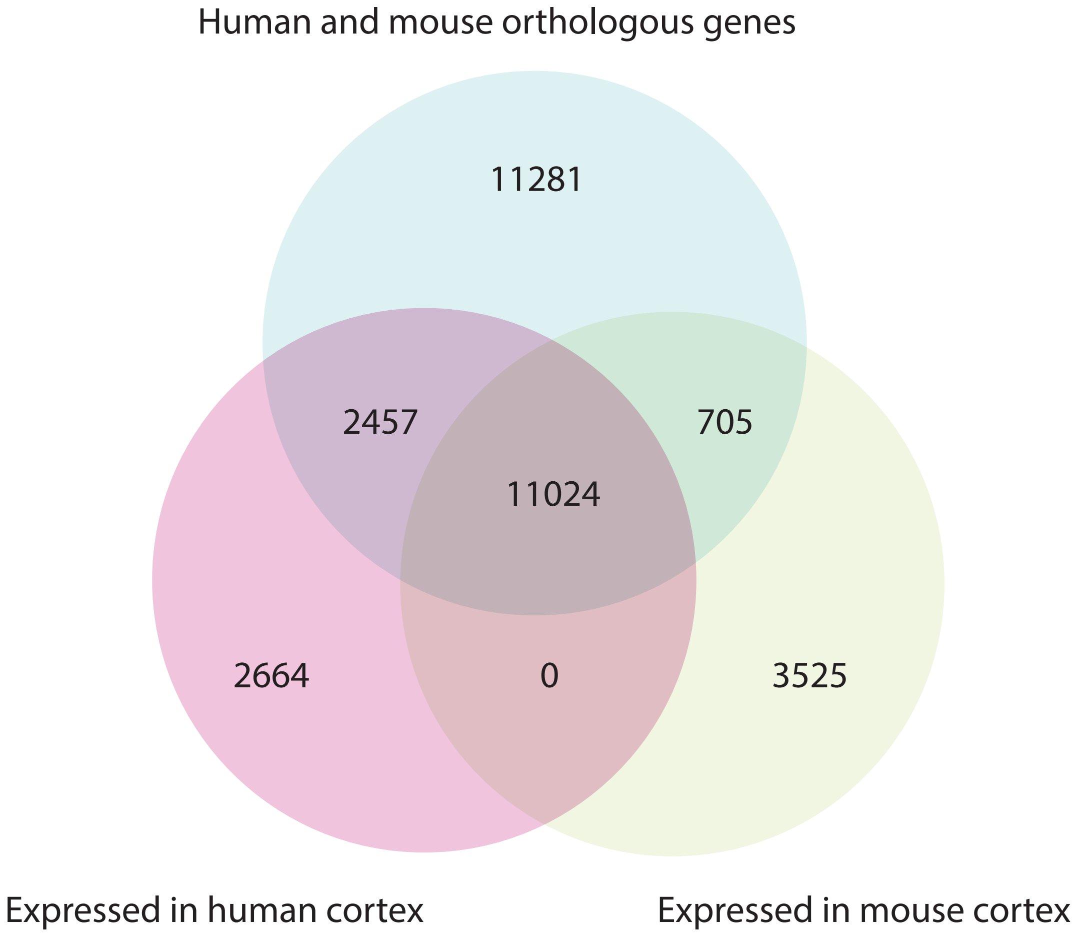

Supplement: Supplementary file 1 [file Data_Sheet_1.zip › Supplementary Figures.pdf]
