# Supplementary material for: Spatially Resolved Transcriptomes of Mammalian Kidneys Illustrate the Molecular Complexity and Interactions of Functional Nephron Segments
Source: Front Med (Lausanne). 2022 Jul 7;9:873923. doi: 10.3389/fmed.2022.873923 (PMC9300864; doi:10.3389/fmed.2022.873923)
Supplement: Supplementary file 1 [file Data_Sheet_1.zip › Supplementary Material.pdf]

10.3389/fmed.2022.873923

Supplementary Material

Supplementary Figure 1. Annotation of functional structures within patients A to C. (A) H&E image and a zoomed-in region of interest (ROI) of the pathologist's annotation of glomeruli and large vasculature. **(B)** The spatial organization of the KNN clusters are mapped to the H&E tissue images, presented in a UMAP and a zoomed-in ROI of clustering. **(C)** The spatial organization of the consensus-based label transfer results are mapped to the H&E tissue images, in a UMAP, a simple bar chart and a zoomed-in ROI of consensus-based label transfer. **(D)** Further deconvolution demonstrates the distribution and proportions of functional structures within the cortical kidney tissue which are mapped to the H&E images, presented as a simple pie chart and a zoomed-in ROI.

Supplementary Figure 2. Quality metrics application in the mouse kidney ST-seq datasets. (A) A linear relationship is demonstrated by scatter plots of the captured genes (y-axis) against reads (x-axis) per ST-spot within the mouse kidney tissue sections (one black dot denotes one ST-spot). **(B)** Violin plots demonstrated that the percentage of mtRNA captured per ST-spot was below 20% in the mouse kidney tissue sections (one black dot denotes one ST-spot). **(C)** The captured percentage mtRNA are spatially mapped to the H&E tissue images (blue spots have captured <10% mtRNA and red spots have captured >15% mtRNA).

Supplementary Figure 3. Quality metrics application in the human cortical kidney ST-seq datasets. (A) H&E images of the human (patient A-D) cortical kidney tissue sections demonstrated normal kidney parenchyma. **(B)** A linear relationship is demonstrated by scatter plots of the captured genes (y-axis) against reads (x-axis) per ST-spot within the cortical kidney tissue sections for patients A-D (one black dot denotes one ST-spot). **(C)** Violin plots of the percentage of mtRNA captured per ST-spot for patients A-D showed that some ST-spots captured mtRNA greater than 50%. The red dashed line indicates the 50% threshold that was used to filter ST-spots with high mtRNA. **(D and E)** Spatial visualization of mtRNA percentage values before **(D)** and after **(E)** filtering (the numbers of ST-spots filtered from each patient ST-seq dataset is noted).

Supplementary Figure 4. Label transfer and annotation of the mouse ST-seq datasets. (A) Decision making hierarchy for the label transfer and annotation of the mouse ST-seq datasets. We performed label transfer in two sequential steps using publicly available mouse kidney scRNA-seq datasets. First, label transfer annotation from both scRNA-seq datasets was used to determine the same high-confidence ST-spot annotations. In the second round, the scRNA-seq datasets were used to label the "unknown" ST-spots with one high-confidence annotation. In both rounds, transfer of cell type annotations from the reference to a query ST-spot was made if the confidence score for the top match was greater than 0.6; remaining ST-spots were left unannotated. Consensus-based label transfer **(B)** using published mouse kidney scRNA-seq (Park et al. 2018; **(C)** - all hits and **(D)** - 0.6 threshold; Miao et al. 2021; **(E)** - all hits and **(F)** - 0.6 threshold) datasets as references in a sequential manner following a decision-making hierarchy.

Supplementary Figure 5. Label transfer and annotation of the human ST-seq datasets. (A) Decision making hierarchy. We performed label transfer in two sequential steps using publicly available human kidney snRNA-seq and scRNA-seq datasets. First, label transfer annotation from the snRNA-seq dataset was used to determine high-confidence ST-spot annotations. In the second round, the scRNA-seq dataset was used to label the remaining unlabeled ST-spots. In both rounds, transfer of cell type annotations from the reference to a query ST-spot was made if the confidence score for

the top match was greater than 0.6; remaining ST-spots were left unannotated. Label transfer within the human ST-seq datasets. Consensus-based label transfer **(B)** using published human kidney snRNA-seq (Blue B. Lake et al. 2019; **(C)** - all hits and **(D)** - 0.6 threshold) and scRNA-seq (Liao et al. 2020; **(E)** - all hits and **(F)** - 0.6 threshold) datasets as references in a sequential manner following a decision-making hierarchy.

Supplementary Figure 6. Orthologous cortical kidney genes. To perform the DE gene analysis between species, we selected cortical kidney regions within the mouse ST-seq datasets and identified mouse-human. This orthology conversion identified 11,997 orthologous genes in the mouse ST-seq datasets. But only 11,024 orthologous genes were identified with one:one ortholog conversion and being expressed in both the mouse and human cortical kidney regions. The orthology conversion did not identify orthologs for 3,525 genes expressed in our mouse cortical kidney regions and 2,664 genes expressed in the human kidneys. Additionally, 11,281 orthologous genes were not expressed in either the mouse and human cortical kidney regions which implied that these orthologous genes may originate in other organs.

Supplementary Table 1. Breakdown of functional units in Miao et al. 2021 and Park et al. 2018 reference datasets used for the annotation by label transfer of the mouse ST-seq datasets.

Functional unit	Miao et al. 2021		Park et al. 2018	
	Counts	Percentage	No. of spots	Counts
Collecting duct	4201	9.6%	2709	6.2%
Distal convoluted tubule	9075	20.8%	8544	19.5%
Endothelial cells	2395	5.5%	n/a	n/a
Fibroblasts	n/a	n/a	549	1.3%
Glomeruli	982	2.3%	78	0.2%
Immune cells	1867	4.3%	2158	4.9%
Loop of Henle	1748	4.0%	1581	3.6%
Nephron progenitors	633	1.5%	n/a	n/a
Proliferating cells	2177	5.0%	n/a	n/a
Proximal tubule	15066	34.5%	26482	60.5%
Stroma	5266	12.1%	n/a	n/a
Unknown	226	0.5%	1644	3.8%
**TOTAL**	**43636**		**43745**	

Supplementary Table 2. Breakdown of the functional units in Blue B. Lake et al. 2019 and Liao et al. 2020 reference datasets used for the annotation by label transfer of the ST-seq datasets.

Functional unit	Blue B. Lake et al. 2019		Liao et al. 2020	
	Counts	Percentage	Counts	Counts
Collecting Duct	4237	25.1%	227	1.0%
Connecting Tubule	395	2.3%	n/a	n/a
Distal Convoluted Tubule	568	3.4%	406	1.7%
Glomeruli	1418	8.4%	606	2.6%
Immune Cells	88	0.5%	1785	7.6%
Interstitium	410	2.4%	n/a	n/a
Loop of Henle	4976	29.5%	n/a	n/a
Proximal Tubule	4587	27.2%	20342	87.1%
Vessels	168	1.0%	n/a	n/a
**TOTAL**	**16847**		**23366**	

Supplementary Table 3. Mouse marker genes from clustering.
Supplementary_Table_1.xlsx.

Supplementary Table 4. Mouse marker genes from consensus label transfer.
Supplementary_Table_2.xlsx.

Supplementary Table 5. Human marker genes from clustering.

Supplementary_Table_4.xlsx.

Supplementary Table 6. Human marker genes from consensus label transfer.

Supplementary_Table_6.xlsx.

Supplementary Table 7. DE genes between species.

Supplementary_Table_7.xlsx.

Supplementary Table 8. Cell location of the top 20 DE genes between species.

DE genes	Ransick et al. 2019 scRNA-seq mouse	Wu et al. 2018 scRNA-seq human	Wu et al. 2019 sc/snRNA-seq mouse
AC011005.1	absent	absent	absent
F13B	PT S2 and S1	DCT	PT S3, S1 and S2
ACSM1	PT S3	absent	PT S3, S1 and S2
CELA1	PT S1 and S2	DCT and LOH	PT S3, S1 and S2
NUDT4B	absent	absent	absent
AC136616.1	absent	absent	absent
MATR3	P and PE	CD PC	EC, MC and P
PDZD9	low expression	LOH	CD IC, CNT and P
CA14	absent	PT and P	absent
DIABLO	P and PE	PT and DCT	MC and P
SPC25	PT S2 and S1	PT	CNT
ATP4A	pCT and pCD	absent	CNT and DCT
DLEC1	LOH(asc), MD, DCT, nCT, pCT and pCD	LOH and PT	CNT, DCT and LOH
SMIM11B	absent	CD IC	absent
ZBED9	absent	low expression	absent
S100G	pCT and nCT	absent	CNT
THY1	low expression	low expression	PT S3 and CNT
CD46	low expression	CD IC	MC
ANPEP	PT S3	PT	CD IC, LOH and PT S3

Key: PT - S1 to S3- proximal tubule segments 1 to 3, P – podocytes, PE - parietal epithelium, pCT - principal-like cell of nephron connecting tubule, pCD - principal-like cell of cortical collecting duct, LOH (asc) - loop of Henle ascending, MD - macula densa, DCT - distal convoluted tubule, nCT - nephron connecting tubule, LOH - loop of Henle, CD PC - collecting duct principal cells, CD IC - collecting duct intercalated cells, EC - endothelial cells and MC - mesangial cells.

Supplementary Table 9. GO Biological Process terms up-regulated in humans.

Supplementary_Table_9.xlsx.

Supplementary Table 10. GO Biological Process terms up-regulated in mice.

Supplementary_Table_10.xlsx.

Supplementary Table 11. CCI in human ST-seq datasets.

Supplementary_Table_11.xlsx.
